# Supplementary material for: Dysfunctional oxidative phosphorylation shunts branched‐chain amino acid catabolism onto lipogenesis in skeletal muscle
Source: EMBO J. 2020 Jun 3;39(14):e103812. doi: 10.15252/embj.2019103812 (PMC7360968; doi:10.15252/embj.2019103812)
Supplement: Supplementary file 1 — Expanded View Figures PDF [file EMBJ-39-e103812-s001.pdf]

Expanded View Figures

Figure EV1. Skm iTRAQ data. (Related to Fig 1).

Upper panel. GSEA bioinformatics analysis of the quantitative proteomic data (iTRAQ) of hindlimb Skm from wt and Low_{OXPHOS} mice. Set to set graph of 14 significantly altered pathways is shown. 1. Hallmarks of adipogenesis; 2. Go_Mitocondrial signal; 3. Kegg_TCA Cycle 4; Pyruvate Metabolism Reactome; 5. Hallmarks of OXPHOS; 6. Go_ETC; 7. Go_NADPH metabolism; 8. Kegg_Propionate metabolism; 9. Redox system; 10. Kegg_BCAA catabolism; 11. Go_Lipid metabolism; 12. Kegg_Lipid oxidation; 13. NEFA, Tg and ketone bodies; 14. Go_FAD-binding proteins. Size, normalized enrichment score (ES, NES) and *P*-values are reported. Color intensity indicates the overlap between subsets. *Medium panel*. Heat maps of the iTRAQ ratio for proteins related to the TCA cycle and reductive carboxylation, glucose and pyruvate metabolism, lipid synthesis and transport, BCAA catabolism and the redox system. Higher intensities of red or blue color represent higher or lower expression ratios, respectively. *Lower panel*. Cytoscape representation of proteomic data.
Data information: Data are representative of 12 animals/genotype.

© 2020 The Authors

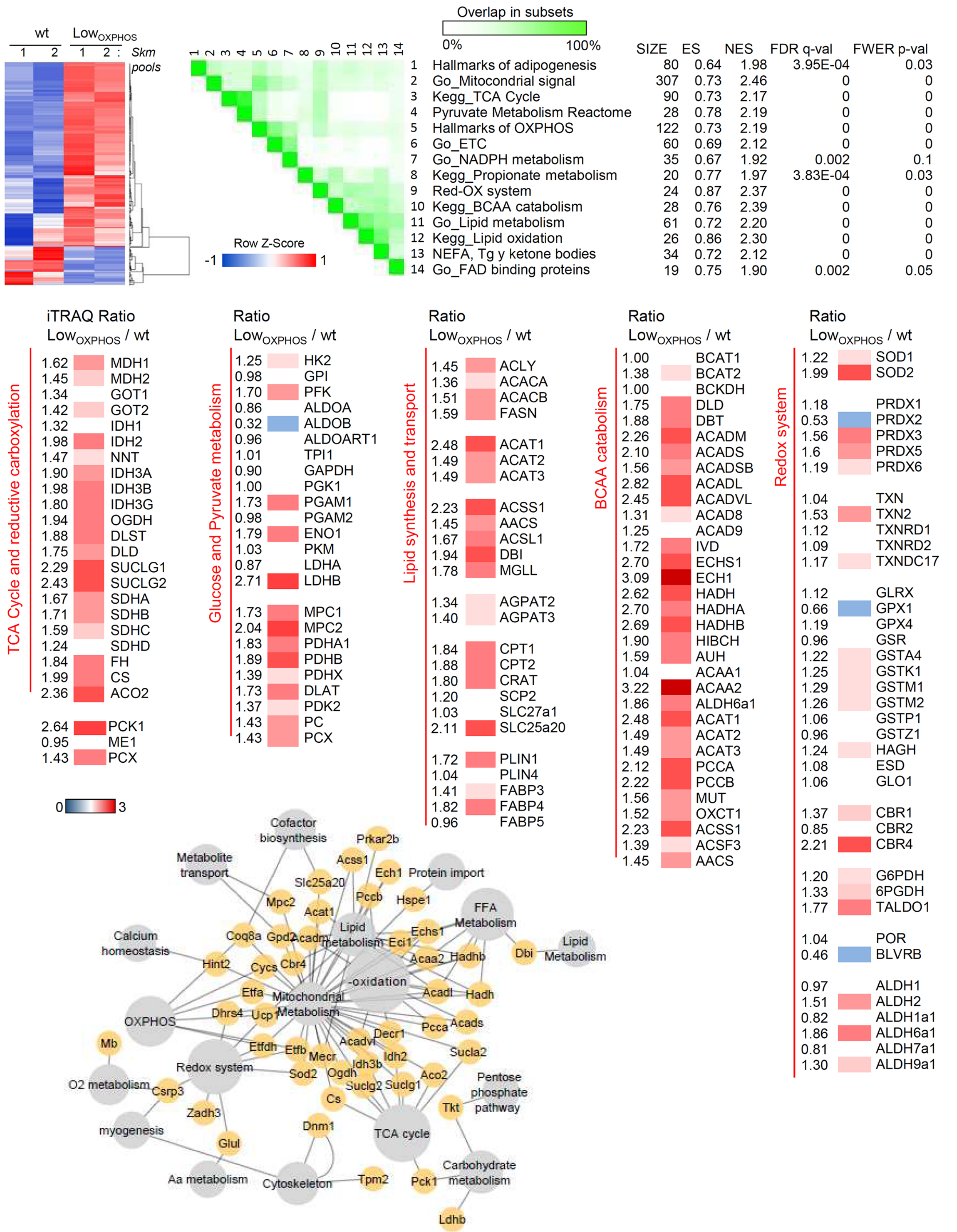

Figure EV1.

 © 2020 The Authors

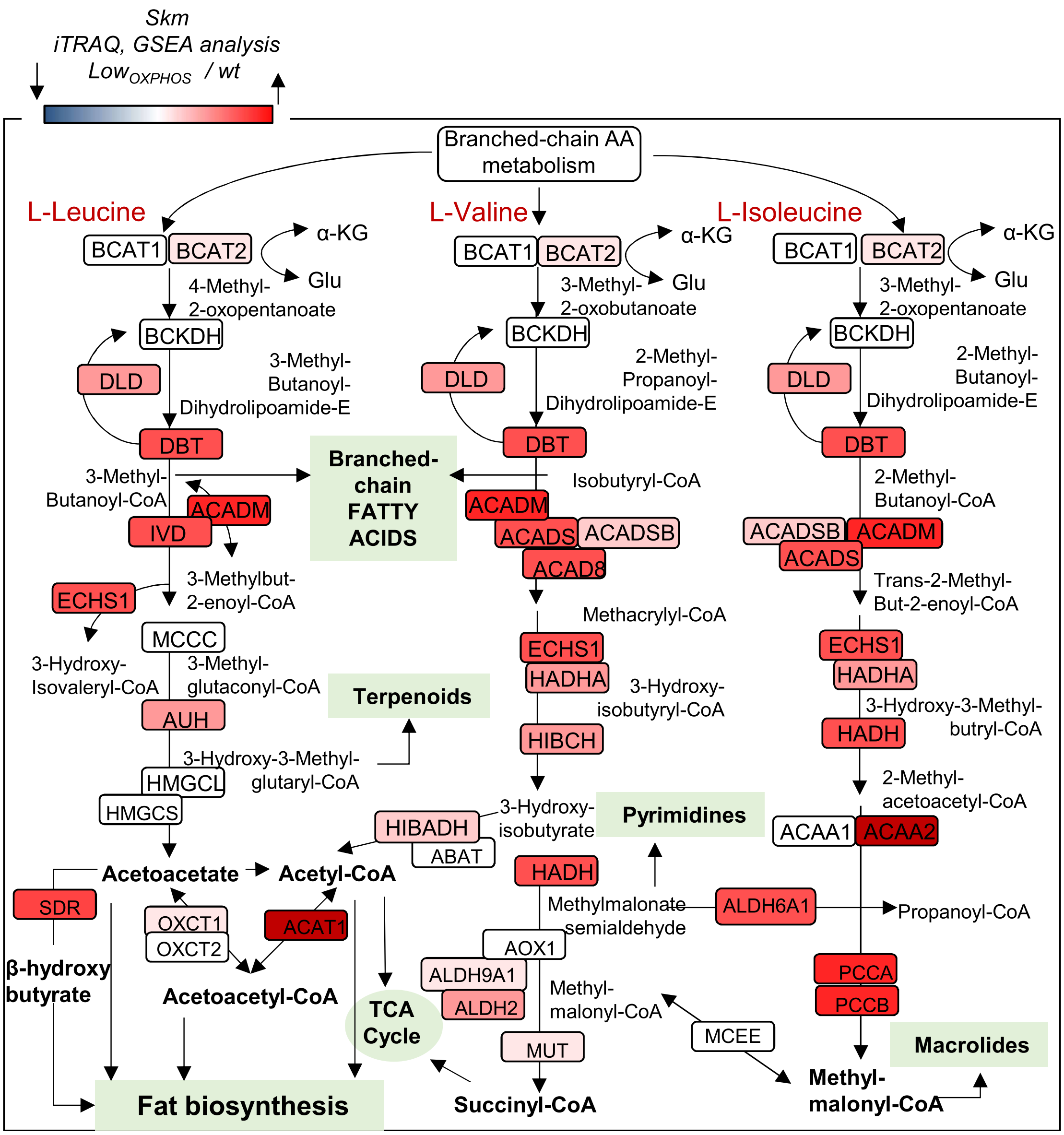

Figure EV2. GSEA analysis of Skm iTRAQ data (BCCA catabolism). (Related to Fig 1).

GSEA representation of the BCAA catabolism pathway obtained by the analysis of the iTRAQ data for hindlimb Skm from wt and Low$_{OXPHOS}$ mice. A higher intensity of red color represents a higher Low$_{OXPHOS}$/wt expression ratio. Data are representative of 12 animals/genotype.

Figure EV3. The ATP synthase-mediated glycolytic and lipogenic reprogramming (related to Figs 2 and 3).

A Representative images of wt and Low_{OXPHOS} mice.
B Representative WB expression of Skm proteins related to glycolysis. LDHA levels are significantly augmented in Low_{OXPHOS} mice when compared to wt. Three samples *per condition* are shown. Each sample contains extracts from 3 mice. Histograms show the LDHA expression (wt, $n = 6$; Low_{OXPHOS}, $n = 6$).
C ATP levels in Skm from wt and Low_{OXPHOS} animals (wt, $n = 5$; Low_{OXPHOS}, $n = 5$). No differences were observed.
D Skm free glycerol levels (wt, $n = 8$; Low_{OXPHOS}, $n = 8$).
E, F LD formation upon ATP synthase inhibition ($ATPIF1_{H49K}$ expression or treatment with 5 μM oligomycin) in C_2C_{12} cells after 24 h of palmitate supplementation. Blue: DAPI, nuclei; green: BODIPY-positive LDs. Images are representative of $n = 3$ experiments, 7–10 fields/condition.
G Representative WB of Skm proteins related with autophagy and inflammation. Two samples *per condition* are shown. Each sample contains extracts from 3 mice (wt, $n = 6$; $Low_{OXPHOS,}$ $n = 6$).
H Representative WB of proteins related with autophagy in C_2C_{12} myocytes expressing or not $ATPIF1_{H49K}$ in the presence or absence of BCAA. No difference where observed. Two samples *per condition* are shown. $n = 3$ experiments, 6 replicates/condition.
I Blood glucose levels after 60 days of doxycycline administration in wt (black bars) and Low_{OXPHOS} (orange bars) mice. No differences were detected between the two genotypes. (wt, $n = 12$; Low_{OXPHOS}, $n = 12$).
J Insulin (ITT) and glucose (GTT) tolerance tests at day 60 of chow diet (wt, $n = 12$; Low_{OXPHOS}, $n = 12$).
K *Upper panel*. Open field representative track plots of wt and Low_{OXPHOS} mice when treated or not with edaravone and fed with HFD. No differences were observed. *Lower panel*. Open field results. Total distance (m), mean speed (m/s), max. speed (m/s), time mobile (s), relative time in the center zone (%) and relative time in the corners (%) of wt (black bars, $n = 4$), wt + edaravone (light green, $n = 4$), Low_{OXPHOS} (orange, $n = 4$) and Low_{OXPHOS} + edaravone (dark green, $n = 4$) are shown.

Data information: Bars are the mean ± SEM of indicated (n) mice. *$P < 0.05$ when compared to wt by Student's t-test.
Source data are available online for this figure.

 © 2020 The Authors

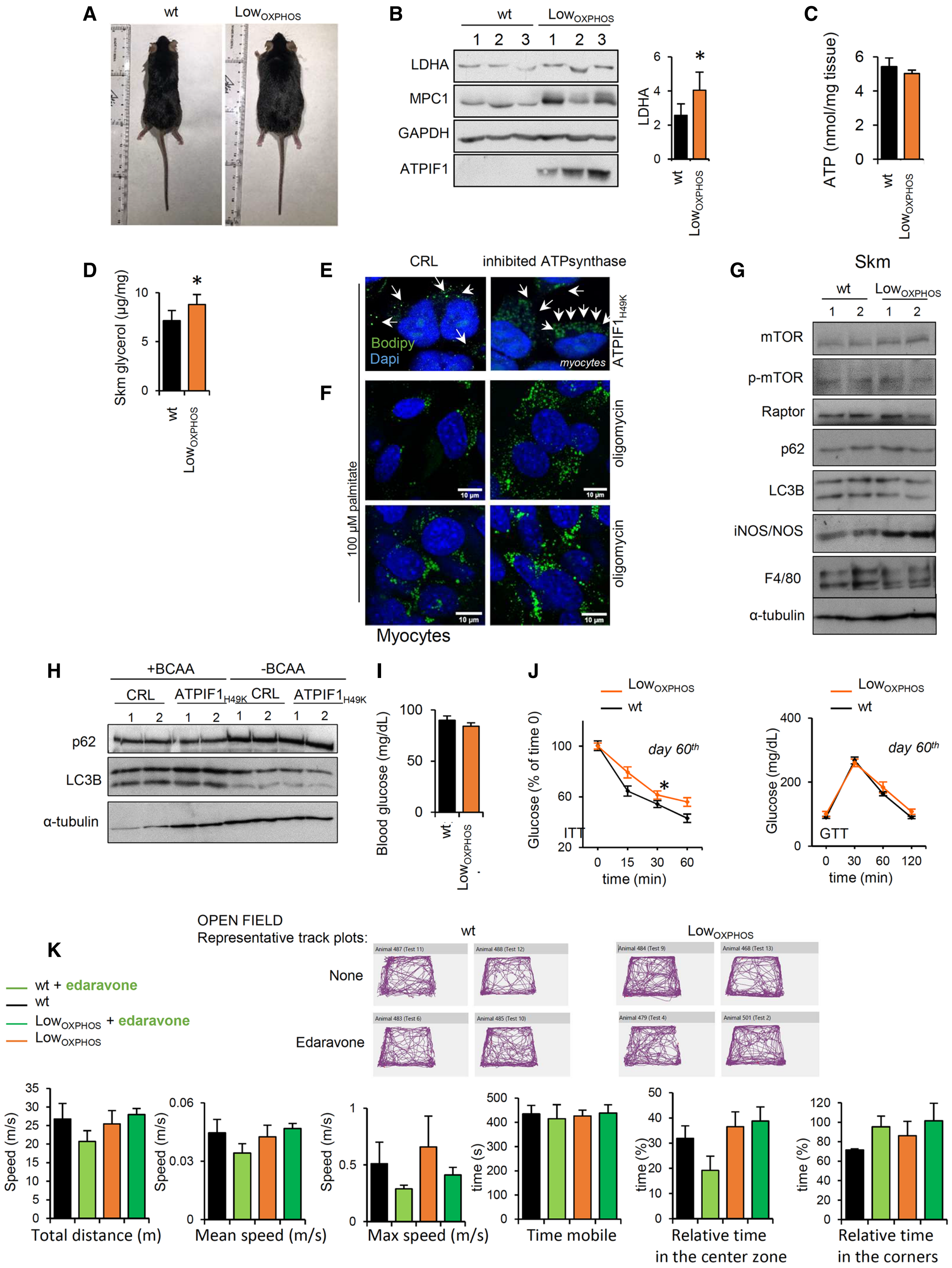
A
wt
Low OXPHOS
B
wt
Low OXPHOS
1 2 3 1 2 3
LDHA
MPC1
GAPDH
ATPIF1
LDHA
wt
Low OXPHOS
C
ATP (nmol/mg tissue)
D
Skm glycerol (µg/mg)
E
CRL
inhibited ATPsynthase
Bodipy
Dapi
myocytes
ATPIF1 H49K
F
100 µM palmitate
oligomycin
Myocytes
G
Skm
mTOR
p-mTOR
Raptor
p62
LC3B
iNOS/NOS
F4/80
α-tubulin
H
+BCAA
-BCAA
CRL
ATPIF1 H49K
I
Blood glucose (mg/dL)
J
day 60th
Glucose (% of time 0)
ITT
time (min)
Glucose (mg/dL)
GTT
K
OPEN FIELD
Representative track plots:
None
Edaravone
wt + edaravone
wt
Low OXPHOS + edaravone
Low OXPHOS
Speed (m/s)
Total distance (m)
Mean speed (m/s)
Max speed (m/s)
time (s)
Time mobile
time (%)
Relative time in the center zone
Relative time in the corners

Figure EV3.

Figure EV4. HFD-dependent alterations in lipid storages and species (related to Figs 3 and 4).

A Weight gained (g/15 days) and mouse body weight following the administration of HFD in wt (black bars and traces, n = 10) and Low_{OXPHOS} (orange bars and traces, n = 10) mice.

B Food intake (g HFD/mouse/day) in wt (n = 5) and Low_{OXPHOS} (n = 4) animals. No differences were observed.

C Representative images of v-WAT in wt and Low_{OXPHOS} mice and total lipids (nmol/mg tissue) in v-WAT from wt (n = 8) and Low_{OXPHOS} (n = 8) animals.

D, E Heat maps of the quantitative lipidomics for TAGs and DAGs species in Skm from wt (n = 8) and Low_{OXPHOS} (n = 8) mice. The color scale (yellow to blue) in the heat map represents the z-score.

F, G WAT saturated DAG (nmol/mg tissue, F) and Skm number of saturated lipid species (G); wt, n = 8; Low_{OXPHOS}, n = 8.

Data information: Data are the mean ± SEM of indicated (n) mice; *$P < 0.05$ when compared to wt by Student's t-test.

© 2020 The Authors

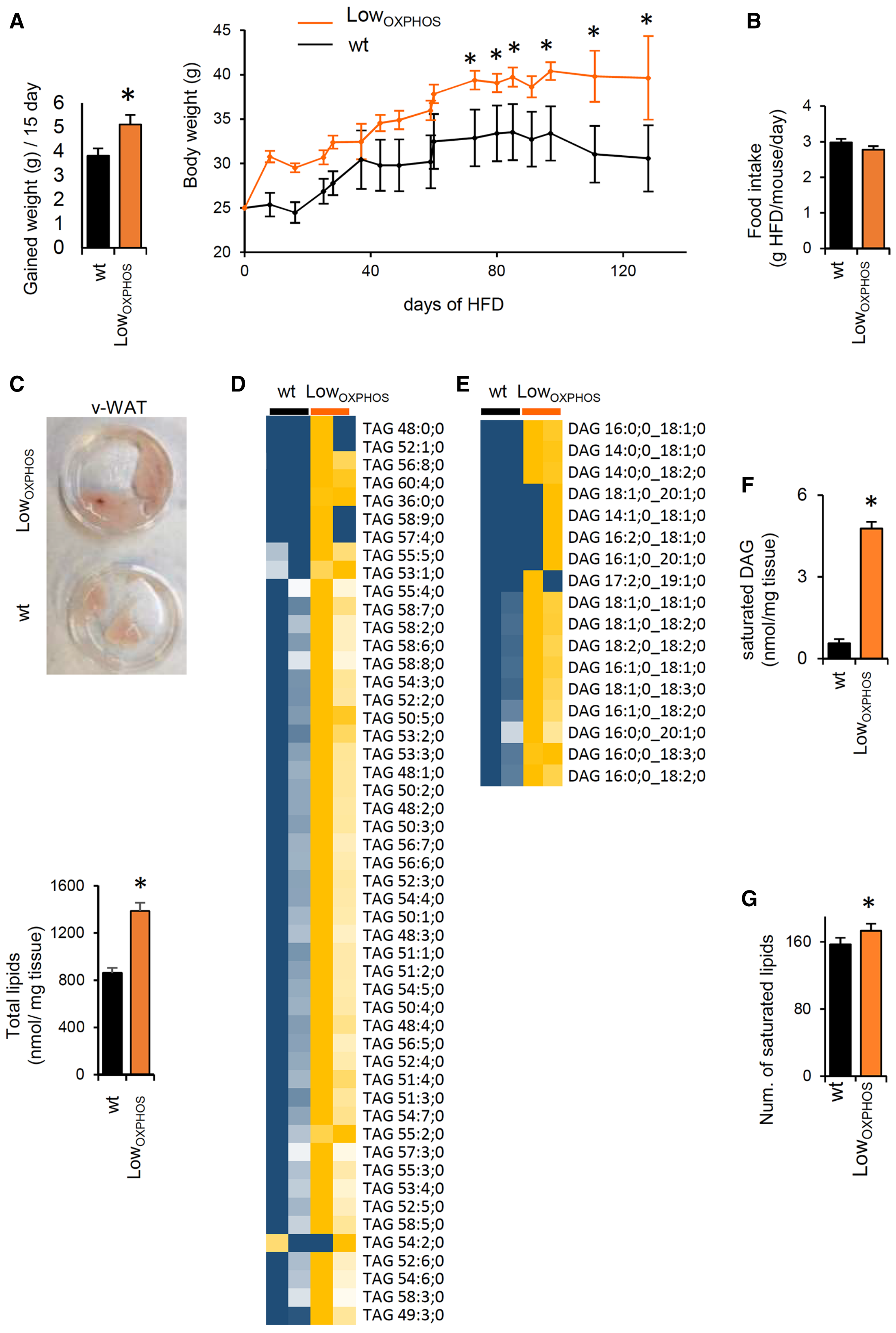
A
Gained weight (g) / 15 day
0
1
2
3
4
5
6
*
wt
Low$_{OXPHOS}$
Body weight (g)
20
25
30
35
40
45
0
40
80
120
days of HFD
Low$_{OXPHOS}$
wt
B
Food intake
(g HFD/mouse/day)
0
1
2
3
4
C
v-WAT
Low$_{OXPHOS}$
wt
Total lipids
(nmol/ mg tissue)
0
400
800
1200
1600
D
wt Low$_{OXPHOS}$
TAG 48:0;0
TAG 52:1;0
TAG 56:8;0
TAG 60:4;0
TAG 36:0;0
TAG 58:9;0
TAG 57:4;0
TAG 55:5;0
TAG 53:1;0
TAG 55:4;0
TAG 58:7;0
TAG 58:2;0
TAG 58:6;0
TAG 58:8;0
TAG 54:3;0
TAG 52:2;0
TAG 50:5;0
TAG 53:2;0
TAG 53:3;0
TAG 48:1;0
TAG 50:2;0
TAG 48:2;0
TAG 50:3;0
TAG 56:7;0
TAG 56:6;0
TAG 52:3;0
TAG 54:4;0
TAG 50:1;0
TAG 48:3;0
TAG 51:1;0
TAG 51:2;0
TAG 54:5;0
TAG 50:4;0
TAG 48:4;0
TAG 56:5;0
TAG 52:4;0
TAG 51:4;0
TAG 51:3;0
TAG 54:7;0
TAG 55:2;0
TAG 57:3;0
TAG 55:3;0
TAG 53:4;0
TAG 52:5;0
TAG 58:5;0
TAG 54:2;0
TAG 52:6;0
TAG 54:6;0
TAG 58:3;0
TAG 49:3;0
E
wt Low$_{OXPHOS}$
DAG 16:0;0_18:1;0
DAG 14:0;0_18:1;0
DAG 14:0;0_18:2;0
DAG 18:1;0_20:1;0
DAG 14:1;0_18:1;0
DAG 16:2;0_18:1;0
DAG 16:1;0_20:1;0
DAG 17:2;0_19:1;0
DAG 18:1;0_18:1;0
DAG 18:1;0_18:2;0
DAG 18:2;0_18:2;0
DAG 16:1;0_18:1;0
DAG 18:1;0_18:3;0
DAG 16:1;0_18:2;0
DAG 16:0;0_20:1;0
DAG 16:0;0_18:3;0
DAG 16:0;0_18:2;0
F
saturated DAG
(nmol/mg tissue)
0
3
6
G
Num. of saturated lipids
0
80
160

Figure EV4.

Figure EV5. Edaravone treatment ameliorates the Skm mitochondrial dysfunctions (related to Figs 4, 5 and 7).

A NADP/NADPH levels in Skm extracts from wt (*n* = 8) and Low$_{OXPHOS}$ (*n* = 8) animals.

B HFD/chow and Low$_{OXPHOS}$/wt iTRAQ ratios of Skm proteins from OXPHOS system (wt, *n* = 10–12; Low$_{OXPHOS}$, *n* = 10–12). Higher intensities of red or blue color represent higher or lower expression ratios, respectively. HFD induced the downregulation of the majority of OXPHOS complex subunits.

C Representative WB of Skm OXPHOS system proteins in mice fed with chow or HFD. Two samples per condition are shown. Each sample contains extracts from 3 mice. NDUFA9 (CI), SDHA and B (CII), ETF subunits (A) and (B), ETFDH, CoreII (CIII), subunit 1 (CIV), βF1(CV), and hATPIF1 immunoblots are shown. Tubulin is provided as a loading control. Histograms show the quantification of specific protein expression (wt, *n* = 6; Low$_{OXPHOS}$, *n* = 6; wt + HFD, *n* = 6; Low$_{OXPHOS}$ + HFD, *n* = 6).

D Enzymatic activity of respiratory complex CI in Skm isolated mitochondria from wt and Low$_{OXPHOS}$ mice fed with chow or HFD (wt, *n* = 4; Low$_{OXPHOS}$, *n* = 4; wt + HFD, *n* = 4; Low$_{OXPHOS}$ + HFD, *n* = 4).

E Representative images of hindlimb muscles of mice treated with edaravone (wt + edaravone, *n* = 4; Low$_{OXPHOS}$ + edaravone, *n* = 4).

F Plasma BCAA levels in wt and Low$_{OXPHOS}$ mice treated or not with edaravone (wt, *n* = 4; Low$_{OXPHOS}$, *n* = 4; wt + edaravone, *n* = 4; Low$_{OXPHOS}$ + edaravone, *n* = 4).

G 14C(u)-leucine uptake in myocytes expressing ATPIF1$_{H49K}$ treated or not with edaravone. Data are the mean ± SEM of *n* = 3 experiments, 6 replicas/condition.

H Food intake (g HFD/mouse/day) in wt and LowOXPHOS animals treated or not with edaravone (wt, *n* = 5; wt + edaravone, *n* = 5). No differences were observed.

Data information: Data are the mean ± SEM of indicated (*n*) mice. $^{*}P < 0.05$ when compared to wt; $^{\#}P < 0.05$ when compared to ATPIF1$_{H49K}$ by ANOVA and Student's *t*-test.
Source data are available online for this figure.

 © 2020 The Authors

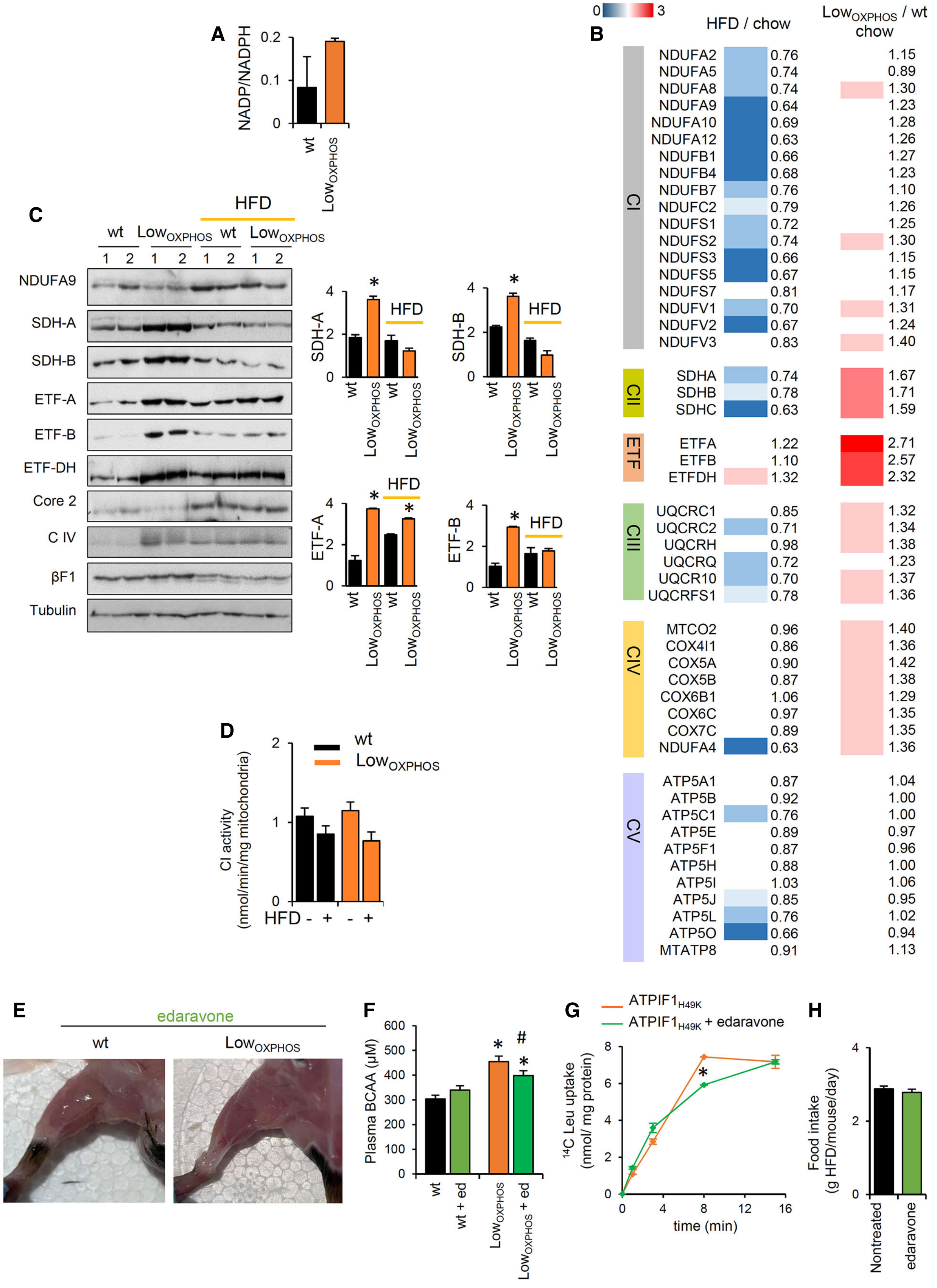
A
NADP/NADPH
0.2
0.1
0
wt
Low$_{OXPHOS}$
B
0
3
HFD / chow
Low$_{OXPHOS}$ / wt chow
CI
NDUFA2 0.76 1.15
NDUFA5 0.74 0.89
NDUFA8 0.74 1.30
NDUFA9 0.64 1.23
NDUFA10 0.69 1.28
NDUFA12 0.63 1.26
NDUFB1 0.66 1.27
NDUFB4 0.68 1.23
NDUFB7 0.76 1.10
NDUFC2 0.79 1.26
NDUFS1 0.72 1.25
NDUFS2 0.74 1.30
NDUFS3 0.66 1.15
NDUFS5 0.67 1.15
NDUFS7 0.81 1.17
NDUFV1 0.70 1.31
NDUFV2 0.67 1.24
NDUFV3 0.83 1.40
CII
SDHA 0.74 1.67
SDHB 0.78 1.71
SDHC 0.63 1.59
ETF
ETFA 1.22 2.71
ETFB 1.10 2.57
ETFDH 1.32 2.32
CIII
UQCRC1 0.85 1.32
UQCRC2 0.71 1.34
UQCRH 0.98 1.38
UQCRQ 0.72 1.23
UQCR10 0.70 1.37
UQCRFS1 0.78 1.36
CIV
MTCO2 0.96 1.40
COX4I1 0.86 1.36
COX5A 0.90 1.42
COX5B 0.87 1.38
COX6B1 1.06 1.29
COX6C 0.97 1.35
COX7C 0.89 1.35
NDUFA4 0.63 1.36
CV
ATP5A1 0.87 1.04
ATP5B 0.92 1.00
ATP5C1 0.76 1.00
ATP5E 0.89 0.97
ATP5F1 0.87 0.96
ATP5H 0.88 1.00
ATP5I 1.03 1.06
ATP5J 0.85 0.95
ATP5L 0.76 1.02
ATP5O 0.66 0.94
MTATP8 0.91 1.13
C
HFD
wt Low$_{OXPHOS}$ wt Low$_{OXPHOS}$
1 2 1 2 1 2 1 2
NDUFA9
SDH-A
SDH-B
ETF-A
ETF-B
ETF-DH
Core 2
C IV
βF1
Tubulin
SDH-A
SDH-B
ETF-A
ETF-B
HFD
*
4
2
0
wt
Low$_{OXPHOS}$
D
CI activity (nmol/min/mg mitochondria)
wt
Low$_{OXPHOS}$
2
1
0
HFD - + - +
E
edaravone
wt
Low$_{OXPHOS}$
F
Plasma BCAA (µM)
600
500
400
300
200
100
0
wt
wt + ed
Low$_{OXPHOS}$
Low$_{OXPHOS}$ + ed
*
#
G
ATPIF1$_{H49K}$
ATPIF1$_{H49K}$ + edaravone
^{14}C Leu uptake (nmol/ mg protein)
8
6
4
2
0
time (min)
0 4 8 12 16
H
Food intake (g HFD/mouse/day)
4
2
0
Nontreated
edaravone

Figure EV5.
