# Supplementary material for: Dysfunctional oxidative phosphorylation shunts branched‐chain amino acid catabolism onto lipogenesis in skeletal muscle
Source: EMBO J. 2020 Jun 3;39(14):e103812. doi: 10.15252/embj.2019103812 (PMC7360968; doi:10.15252/embj.2019103812)
Supplement: Supplementary file 5 — Source Data for Expanded View [file EMBJ-39-e103812-s010.zip › EMBOJ-2019-103812R1-Figure_EV3_uncropped_gels-sd.pdf]

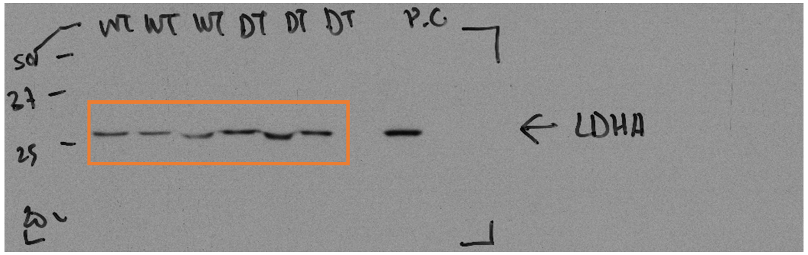

- **Figure EV3B**
- Ab: LDHA
- 27/04/2018

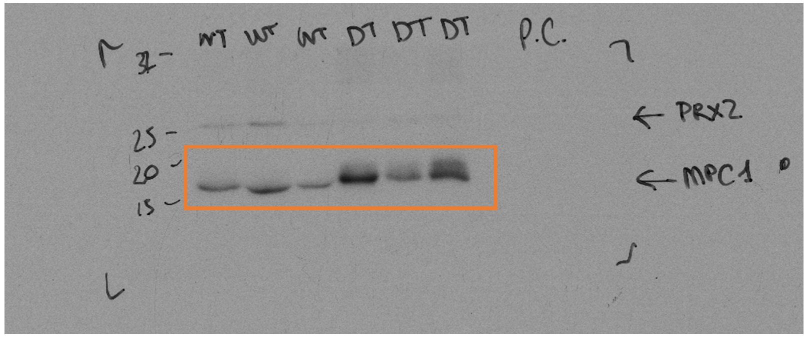

- **Figure EV3B**
- Ab: MPC1
- 12/07/2018

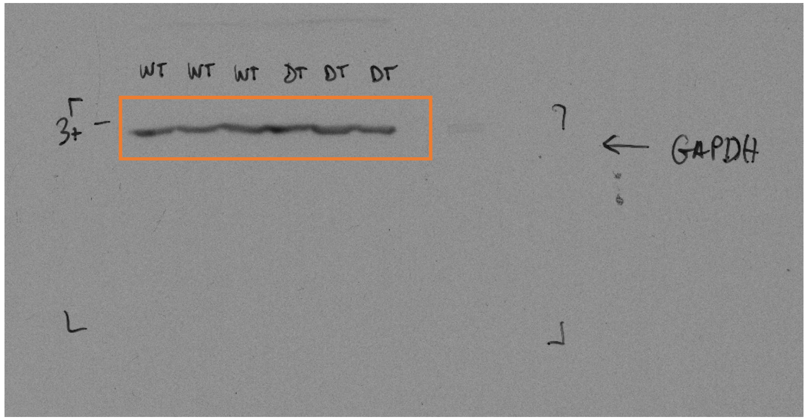

- **Figure EV3B**
- Ab: GAPDH
- 27/06/2018

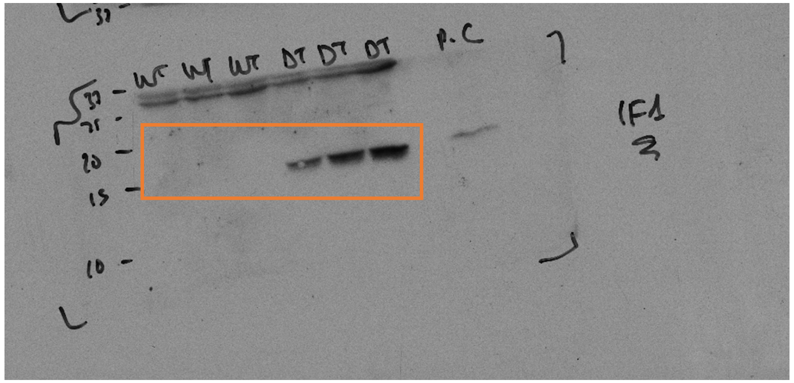

- **Figure EV3B**
- Ab: human ATPIF1
- 09/08/2018

Wt= wt
$ATPIF1_{H49K}$= DT

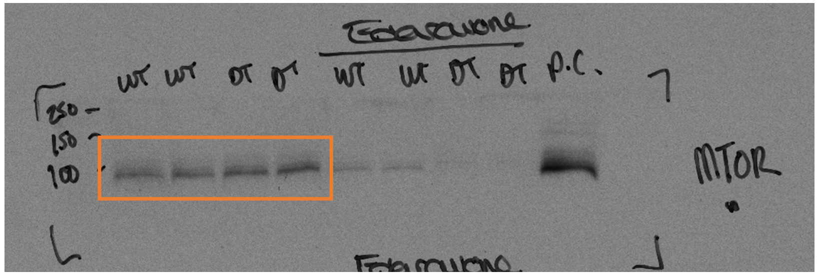

- **Figure EV3G**
- Ab: mTOR
- 15/05/2019

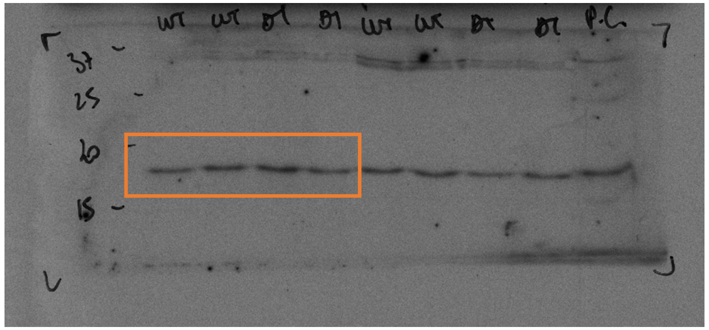

- **Figure EV3G**
- Ab: p62
- 29/11/2019

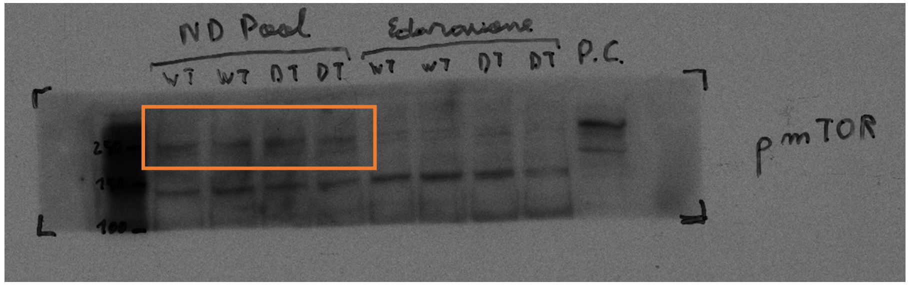

- **Figure EV3G**
- Ab: p-mTOR
- 22/05/2019

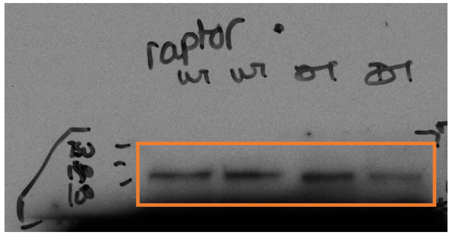

- **Figure EV3G**
- Ab: Raptor
- 10/01/2020

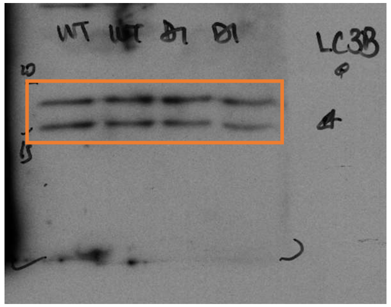

- **Figure EV3G**
- Ab: LC3B
- 10/01/2020

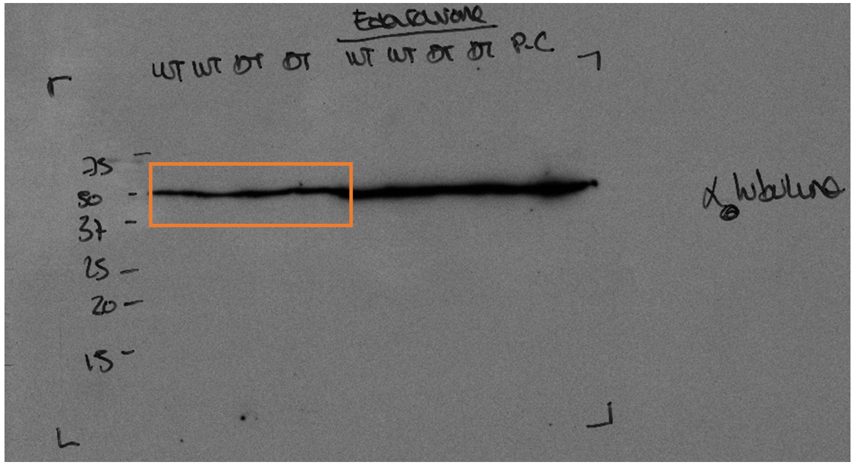

- **Figure EV3G**
- Ab: α tubulin
- 29/11/2019

Wt= wt
ATPIF1$_{H49K}$= DT

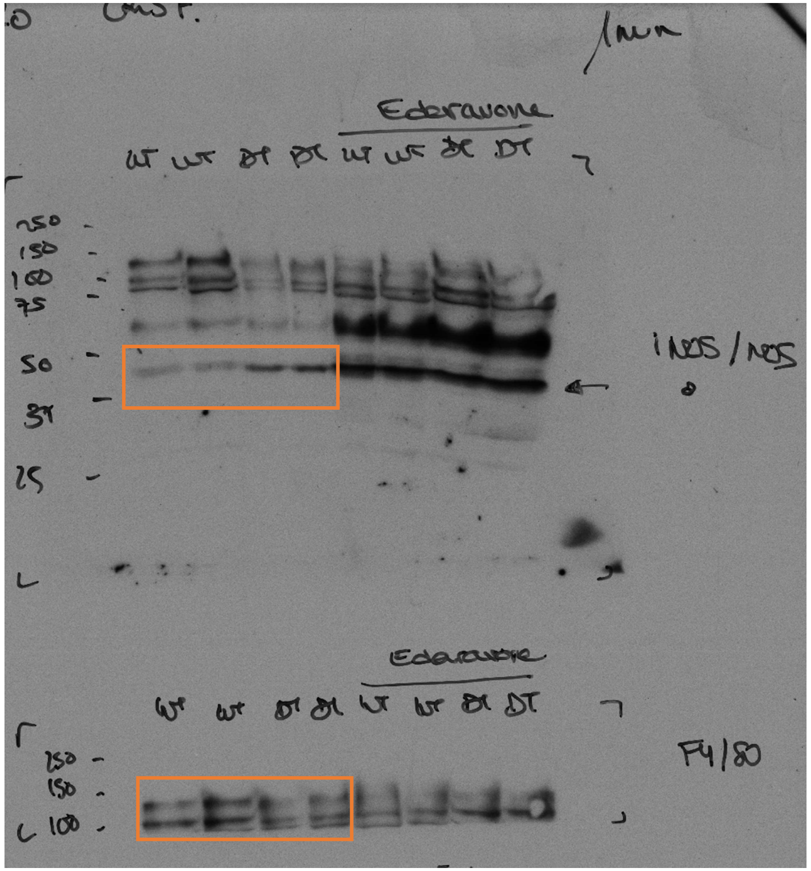

- **Figure EV3G**
- <u>Ab: iNOS/NOS and F4/80</u>
- 10/01/2020

Wt= wt
$ATPIF1_{H49K}$= DT

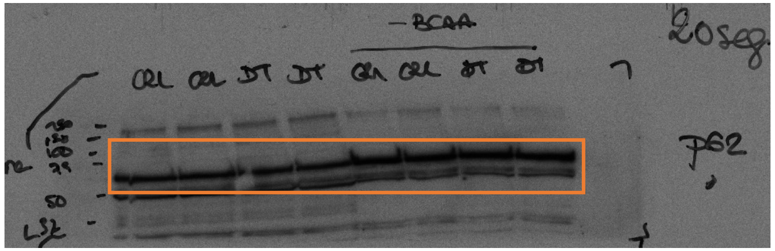

- **Figure EV3H**
- <u>Ab: p62</u>
- 29/11/2019

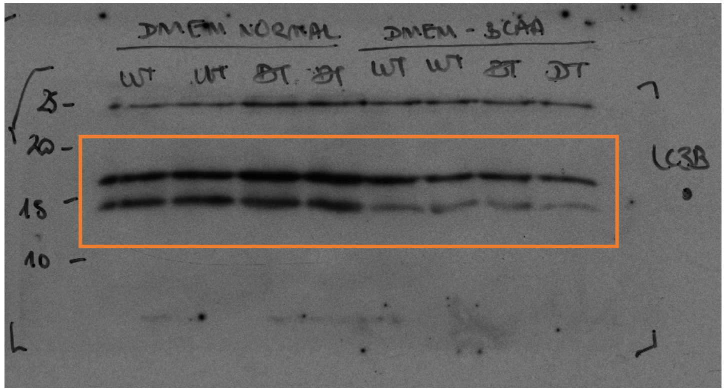

- **Figure EV3H**
- <u>Ab: LC3B</u>
- 10/01/2020

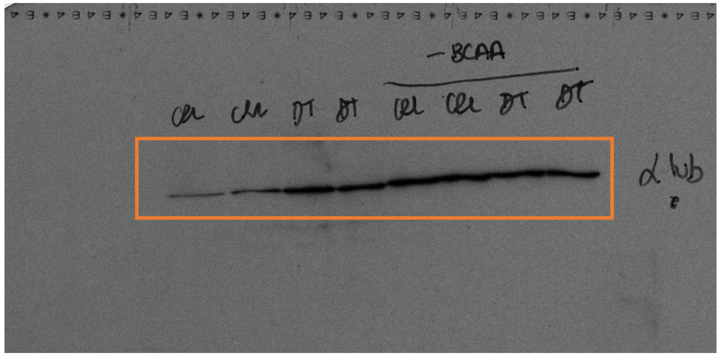

- **Figure EV3H**
- <u>Ab: α tubulin</u>
- 29/11/2019
