# Supplementary material for: Dysfunctional oxidative phosphorylation shunts branched‐chain amino acid catabolism onto lipogenesis in skeletal muscle
Source: EMBO J. 2020 Jun 3;39(14):e103812. doi: 10.15252/embj.2019103812 (PMC7360968; doi:10.15252/embj.2019103812)
Supplement: Supplementary file 5 — Source Data for Expanded View [file EMBJ-39-e103812-s010.zip › EMBOJ-2019-103812R1-Figure_EV5_uncropped_gels-sd.pdf]

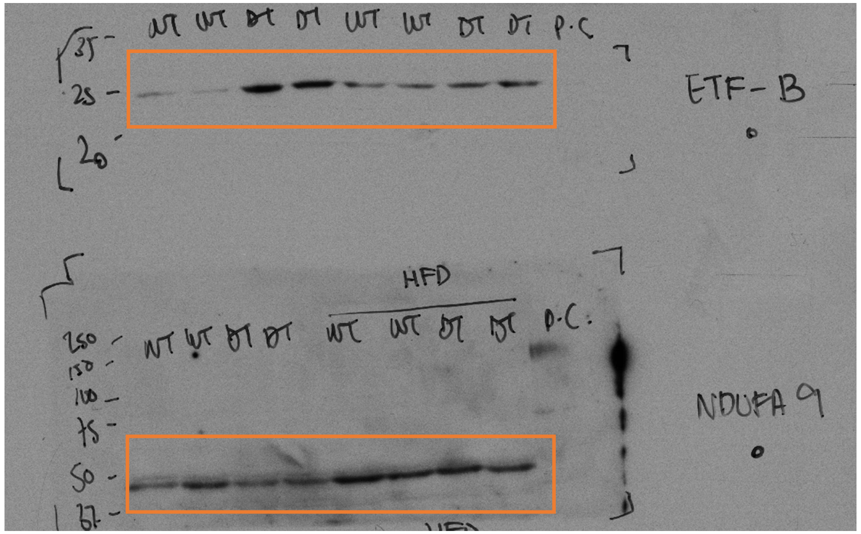

- **Figure EV5C**
- Ab: NDUFA9 and ETFB
- 16/04/2019

- **Figure EV5C**
- Ab: ETFA
- 16/04/2019

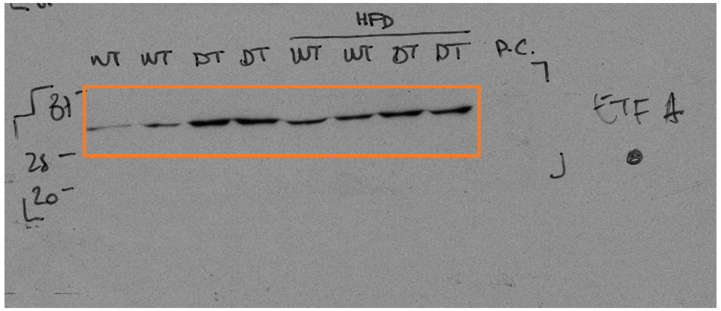

- **Figure EV5C**
- Ab: ETFDH
- 9/07/2019

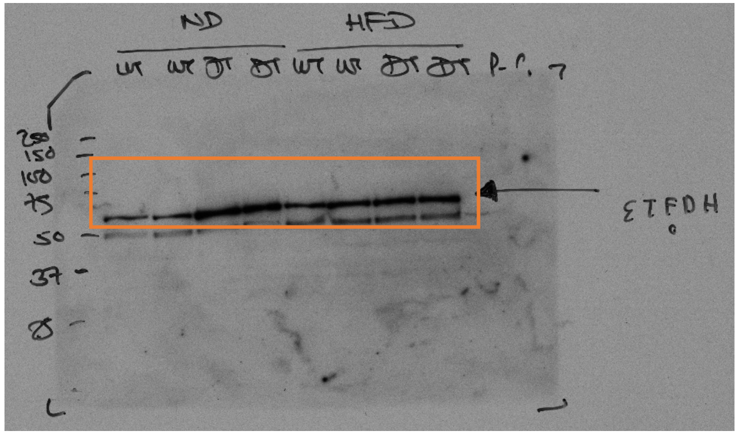

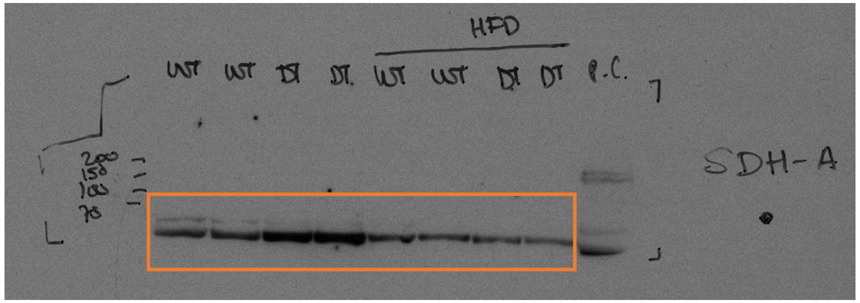

- **Figure EV5C**
- Ab: SDHA
- 16/01/2019

- **Figure EV5C**
- Ab: Core 2
- 17/04/2019

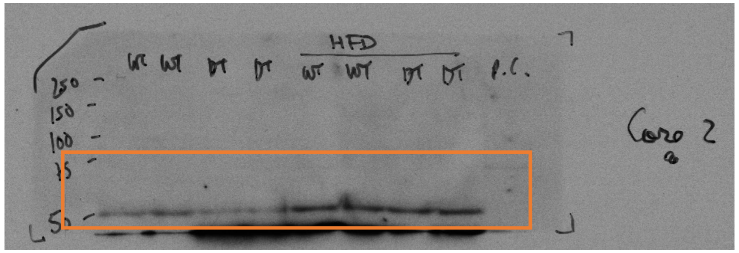

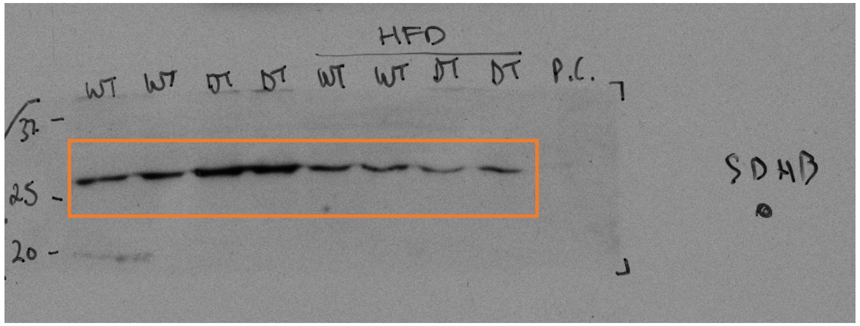

- **Figure EV5C**
- Ab: SDHB
- 17/04/2019

Wt= wt
ATPIF1$_{H49K}$= DT

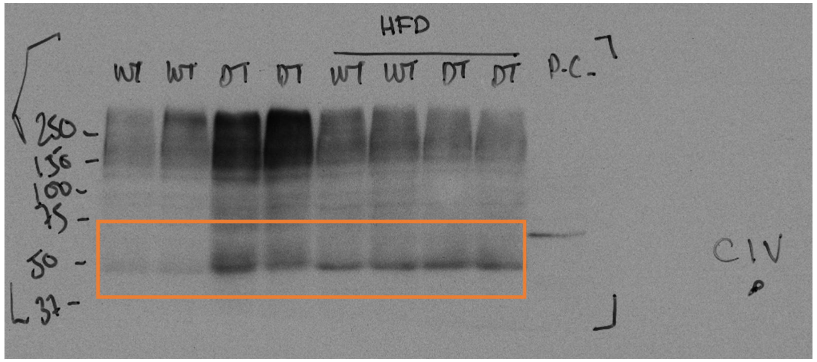

- **Figure EV5C**
- Ab: C IV
- 16/04/2019

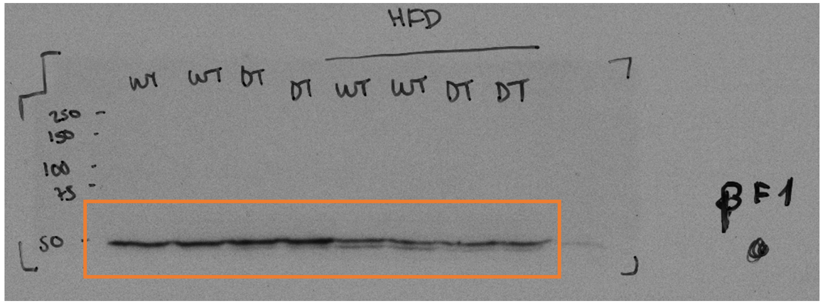

- **Figure EV5C**
- Ab: βF1
- 17/04/2019

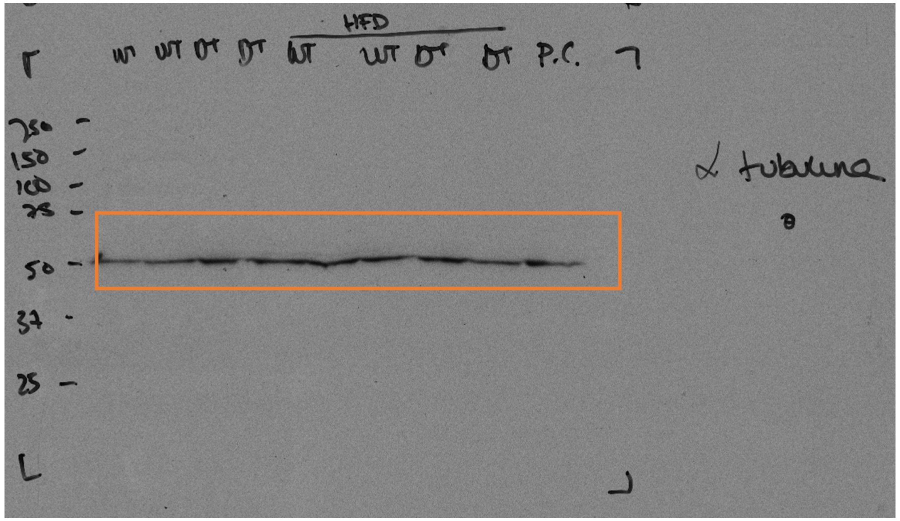

- **Figure EV5C**
- Ab: α tubulin
- 14/05/2019

Wt= wt
$ATPIF1_{H49K}$= DT
