# Supplementary material for: Dysfunctional oxidative phosphorylation shunts branched‐chain amino acid catabolism onto lipogenesis in skeletal muscle
Source: EMBO J. 2020 Jun 3;39(14):e103812. doi: 10.15252/embj.2019103812 (PMC7360968; doi:10.15252/embj.2019103812)
Supplement: Supplementary file 7 — Source Data for Figure 1 [file EMBJ-39-e103812-s005.pdf]

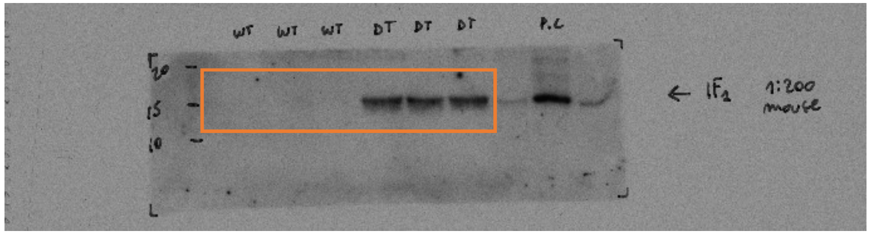

- **Figure 1B**
- Ab: human ATPIF1
- Date: 1/03/2018

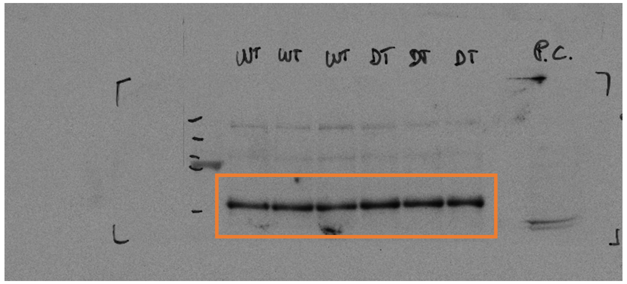

- **Figure 1B**
- Ab: HSP60
- Date: 16/03/2018

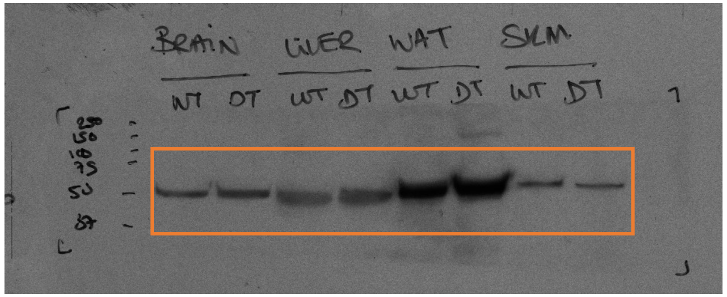

- **Figure 1D**
- Ac: α-tubulin
- Date: 19/12/2019

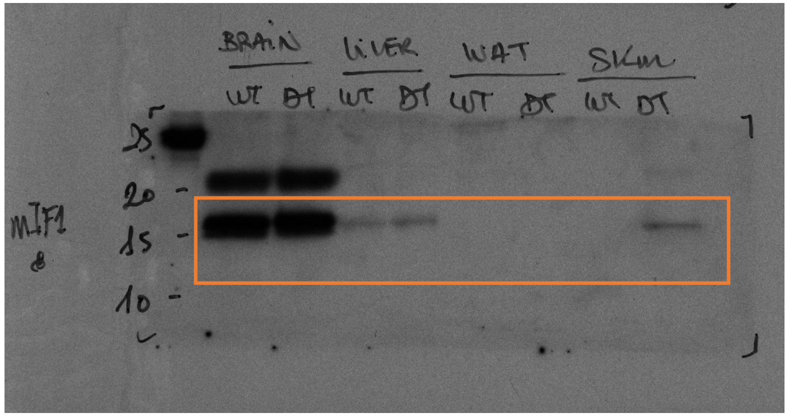

- **Figure 1D**
- Ac: mouse+ human ATPIF1
- Date: 19/12/2019

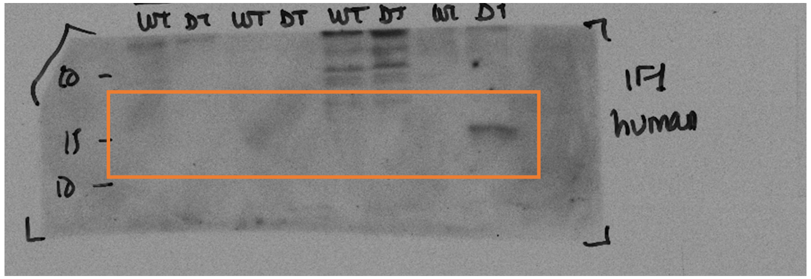

- **Figure 1D**
- Ab: human ATPIF1
- Date: 19/12/2019

Wt= wt
$ATPIF1_{H49K}$= DT
