# Supplementary material for: Dysfunctional oxidative phosphorylation shunts branched‐chain amino acid catabolism onto lipogenesis in skeletal muscle
Source: EMBO J. 2020 Jun 3;39(14):e103812. doi: 10.15252/embj.2019103812 (PMC7360968; doi:10.15252/embj.2019103812)
Supplement: Supplementary file 8 — Source Data for Figure 2 [file EMBJ-39-e103812-s006.pdf]

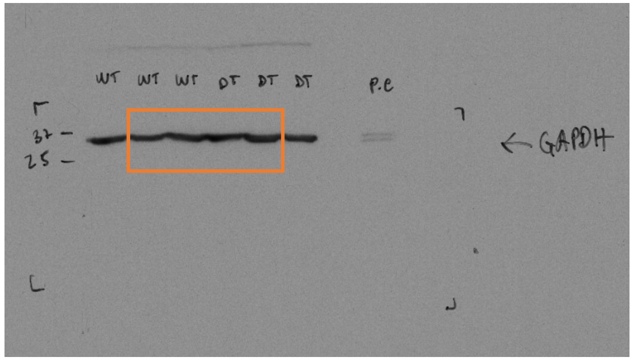

- **Figure 2F**
- Ab: <u>GAPDH</u>
- Date: 18/06/2018

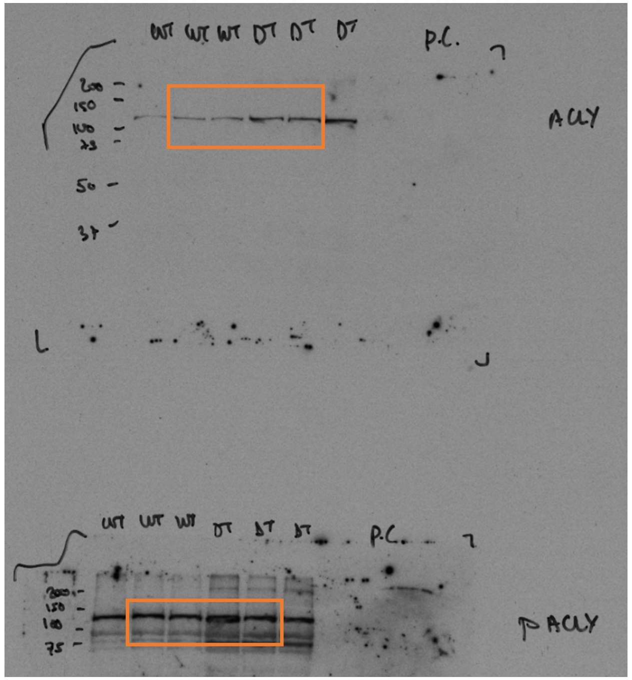

- **Figure 2F**
- Ab: <u>ACLY,</u>
- <u>pACLY</u>
- Date: 11/12/2018

Wt= wt
$ATPIF1_{H49K}$= DT

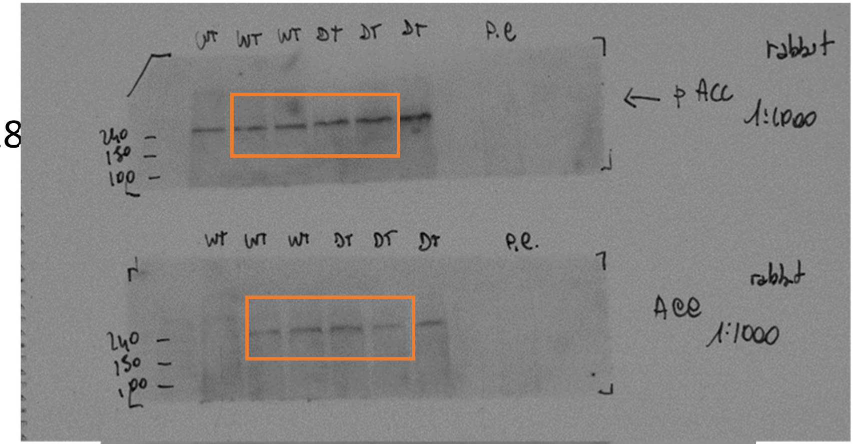

- **Figure 2F**
- Ab: p<u>ACAC,</u>
- <u>ACAC</u>
- Date:12/03/2018

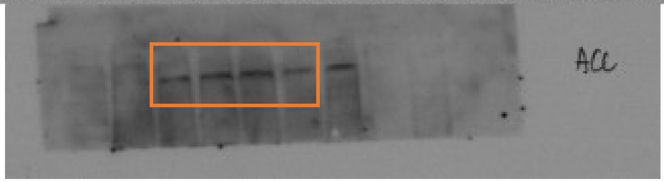

- <u>ACAC, high exposure</u>

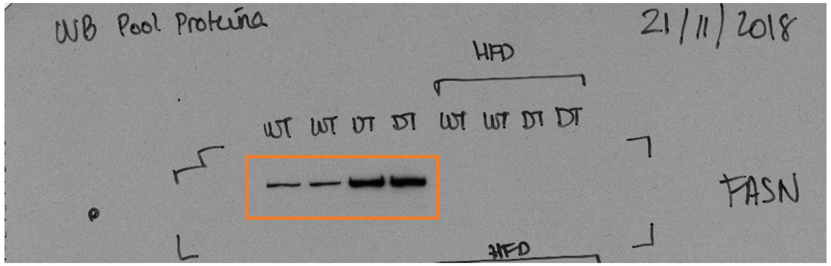

- **Figure 2F**
- Ab: <u>FASN</u>
- Date: 26/11/2018

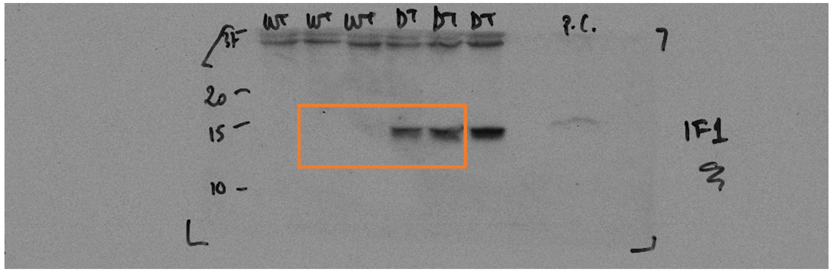

- **Figure 2F**
- Ab: hATP<u>IF1</u>
- Date: 09/08/2018

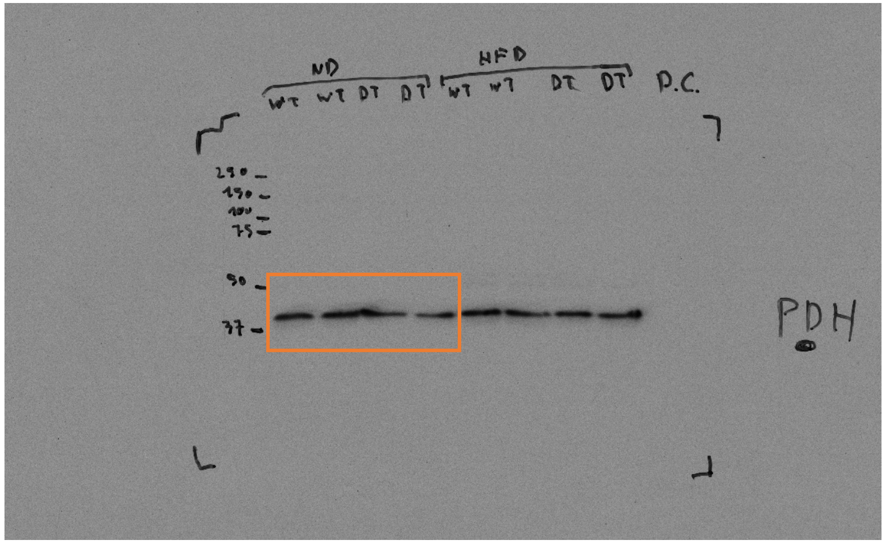

- **Figure 2M**
- Ab: pPDH
- Fecha: 23/07/2019

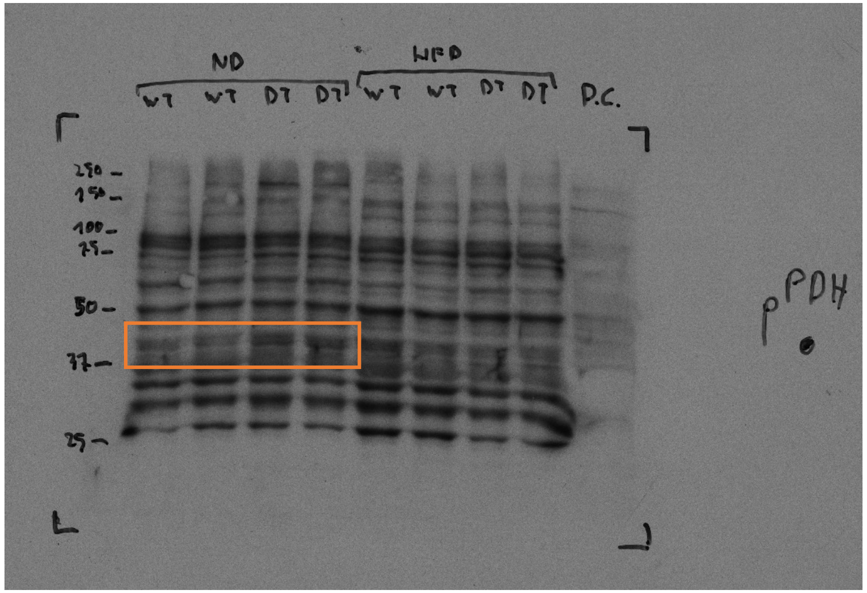

- **Figure 2M**
- Ab: PDH
- Fecha: 23/07/2019

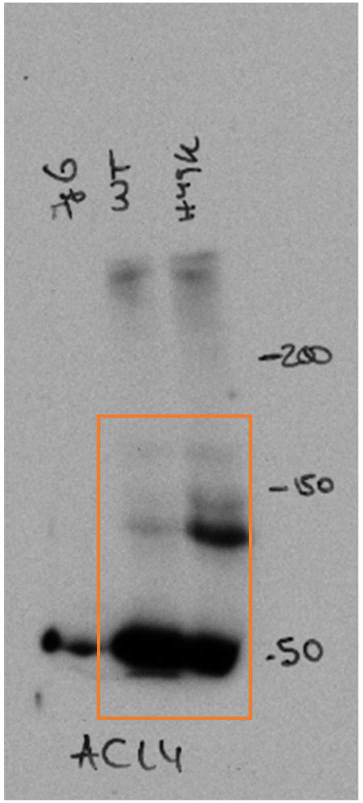

- **Figure 2G**
- IP: Acetyl-K
- WB: ACLY
- Date: 23/01/2020

Wt= wt
ATPIF1$_{H49K}$= DT
