# Supplementary material for: Dysfunctional oxidative phosphorylation shunts branched‐chain amino acid catabolism onto lipogenesis in skeletal muscle
Source: EMBO J. 2020 Jun 3;39(14):e103812. doi: 10.15252/embj.2019103812 (PMC7360968; doi:10.15252/embj.2019103812)
Supplement: Supplementary file 9 — Source Data for Figure 4 [file EMBJ-39-e103812-s007.pdf]

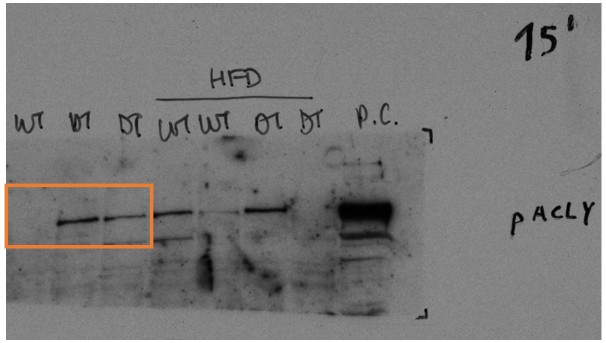

- **Figure 4C**
- Ab: pACLY
- Date: 09/04/2019

- **Figure 4C**
- Ab: human ATPIF1
- Date: 22/03/2019

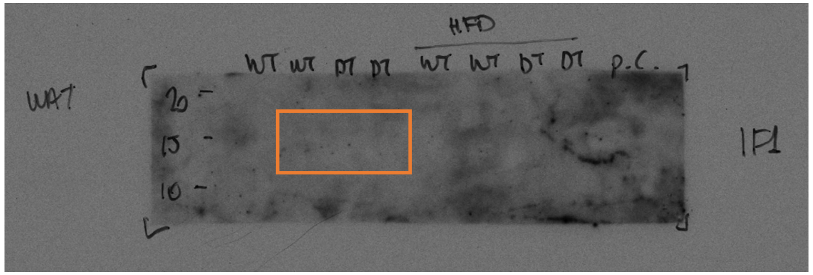

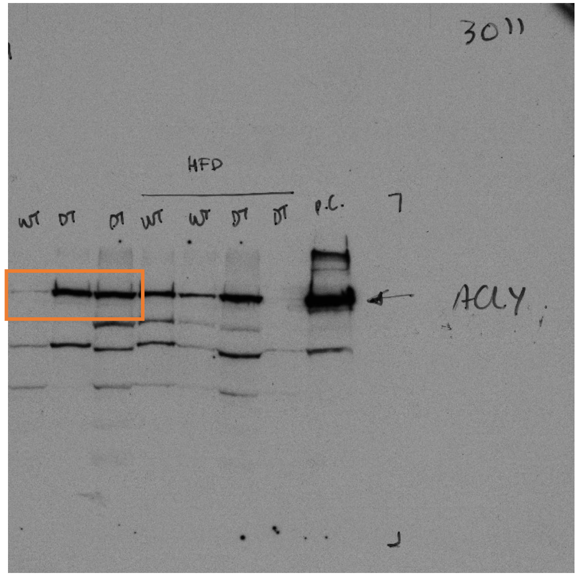

- **Figure 4C**
- Ab: ACLY
- Date: 27/03/2019

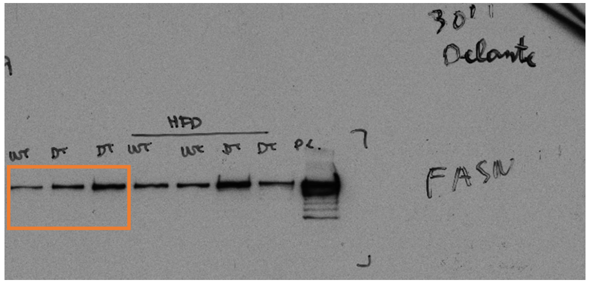

- **Figure 4C**
- Ab: FASN
- Date: 02/04/2019

Wt= wt
$ATPIF1_{H49K}$= DT

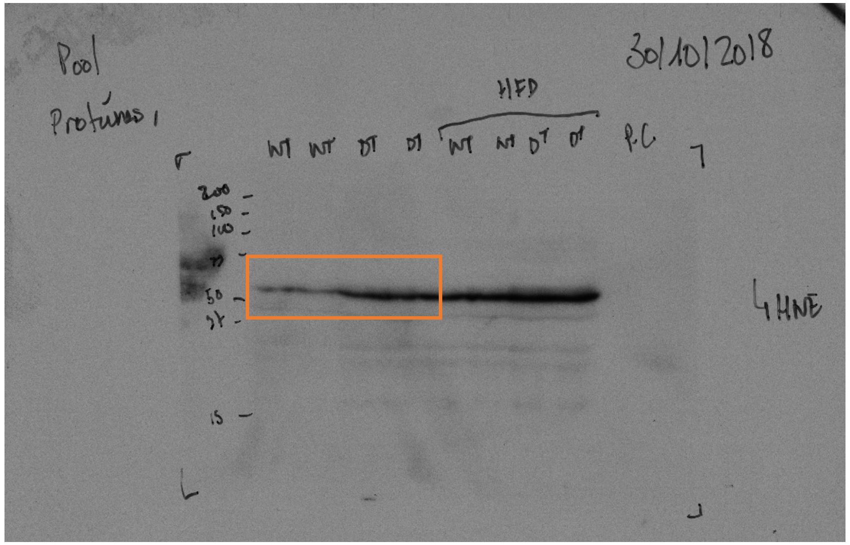

- **Figure 4J**
- Ab: 4HNE
- Date: 30/10/2019

- **Figure 4J**
- Ab: PRX3
- Date: 02/08/2018

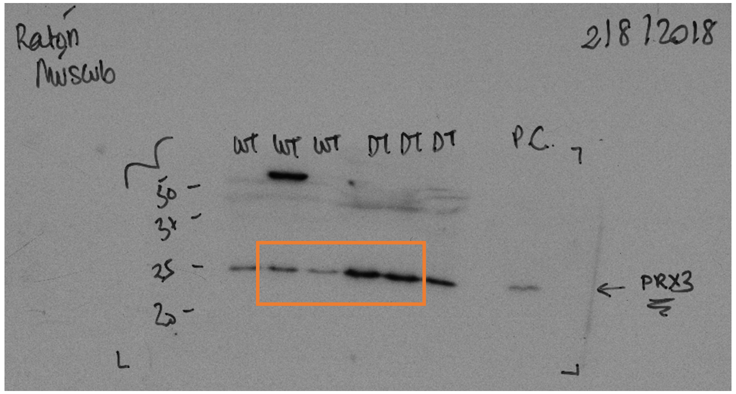

- **Figure 4J**
- Ab: PRX6
- Date: 16/03/2018

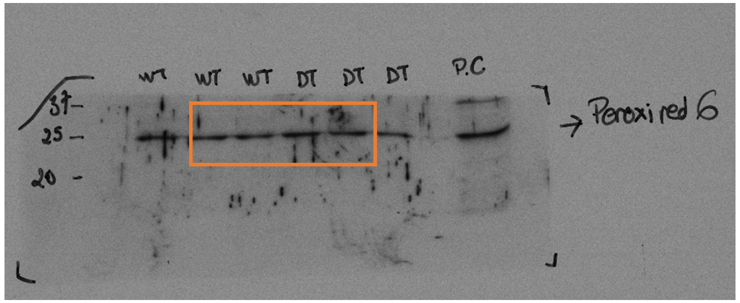

wt wt dt dt wt wt dt dt

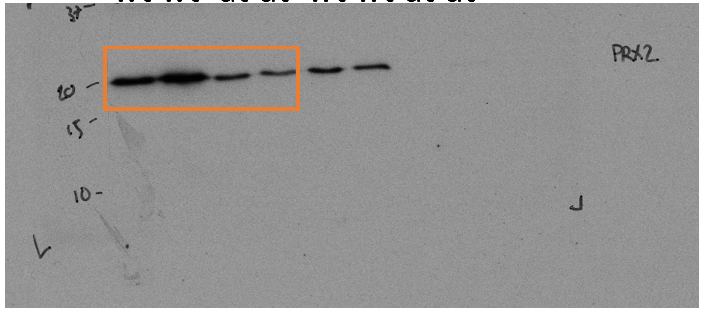

- **Figure 4J**
- Ab: PRX2
- Date: 30/10/2019

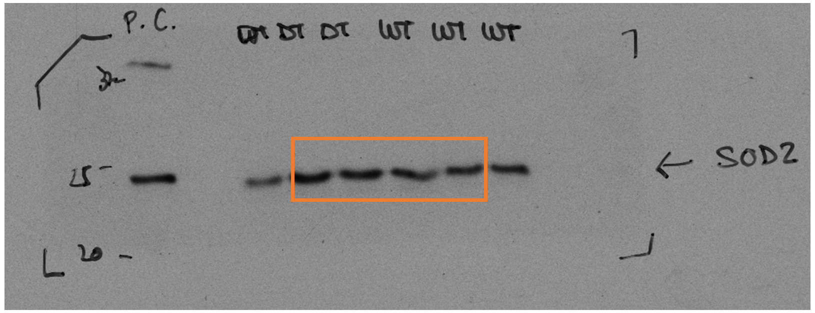

- **Figure 4J**
- Ab: SOD2
- Date: 04/05/2018

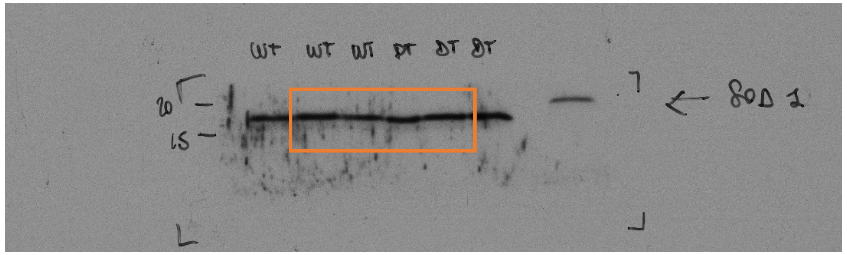

- **Figure 4J**
- Ab: SOD1
- Fecha: 5/07/2018

Wt= wt
ATPIF1$_{H49K}$= DT

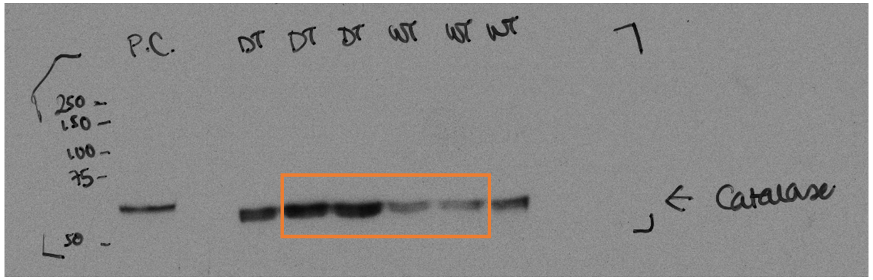

- **Figure 4J**
- <u>Ab: CATALASE</u>
- Date: 04/05/2018

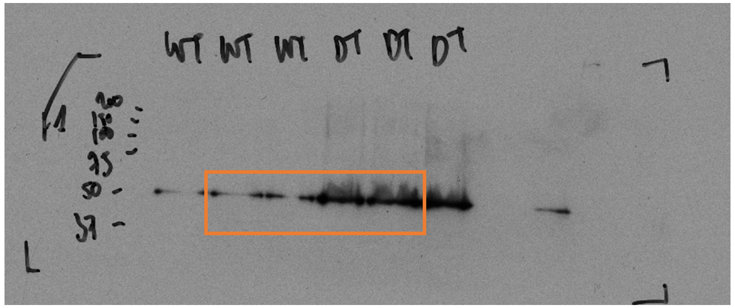

- **Figure 4J**
- <u>Ab: GSR</u>
- Date: 20/12/2018

wt wt wt wt dt dt

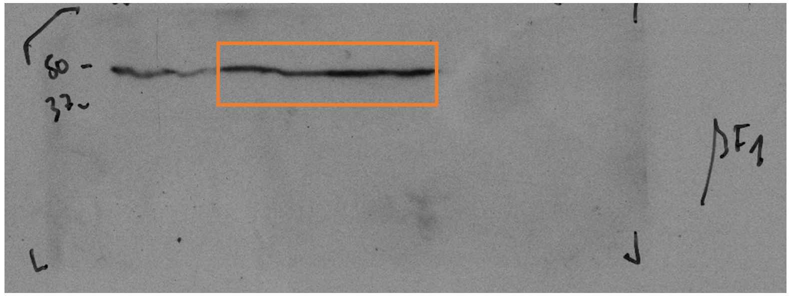

- **Figure 4J**
- <u>Ab: βF1</u>
- Date: 02/08/2018

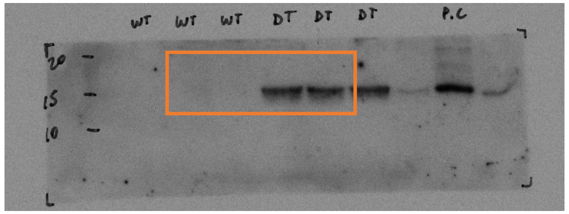

- **Figure 4J**
- <u>Ab: hATPIF1</u>
- Date: 01/03/2018

Wt= wt
$ATPIF1_{H49K}$= DT

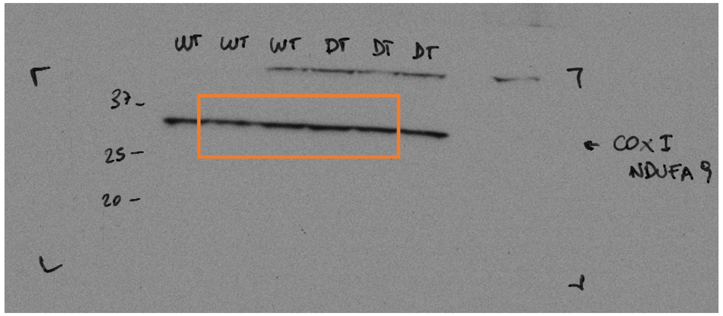

- **Figure 4K**
- Ab: NDUFA9
- Date: 16/03/2018

Wt= wt
ATPIF1$_{H49K}$= DT

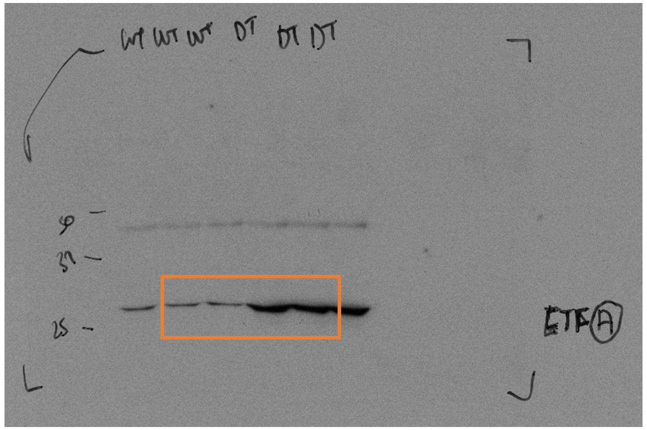

- **Figure 4K**
- Ab: ETFA
- Date: 12/12/2018

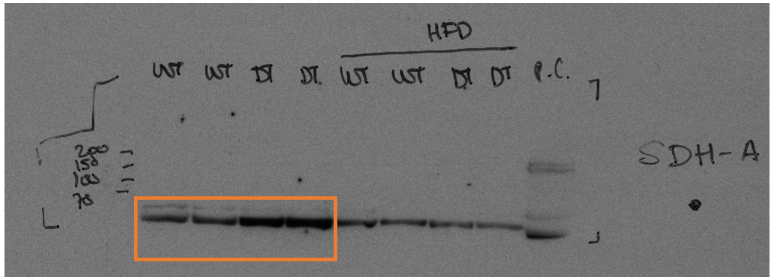

- **Figure 4K**
- Ab: SDHA
- Date: 16/01/2019

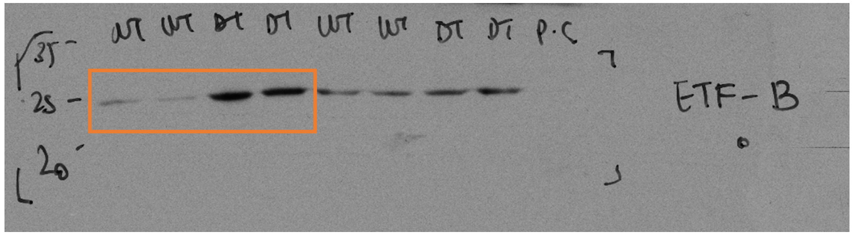

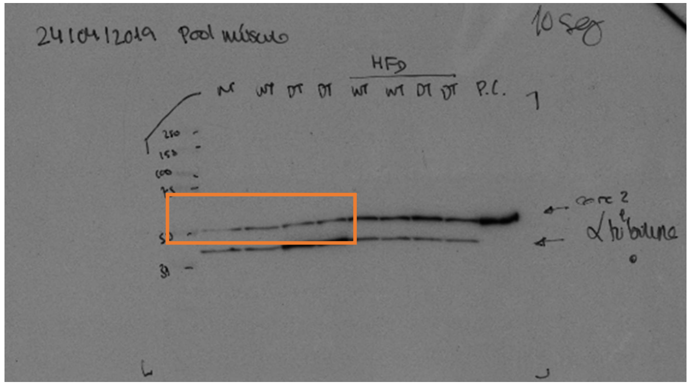

- **Figure 4K**
- Ab: Core II
- Date: 24/04/2019

- **Figure 4K**
- Ab: ETFB
- Date: 16/04/2019

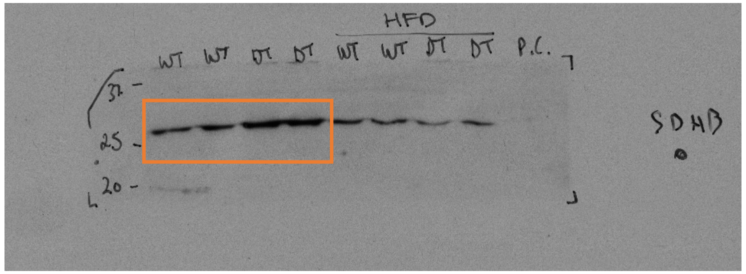

- **Figure 4K**
- Ab: SDHB
- Date: 24/04/2019

- **Figure 4K**
- Ab: ETF-DH
- Date: 09/07/2019

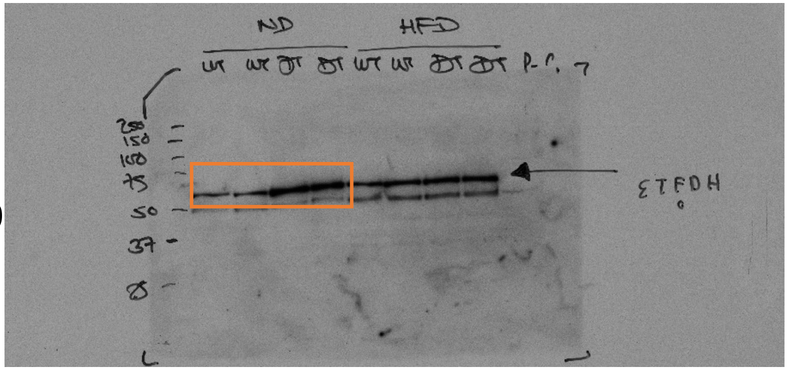

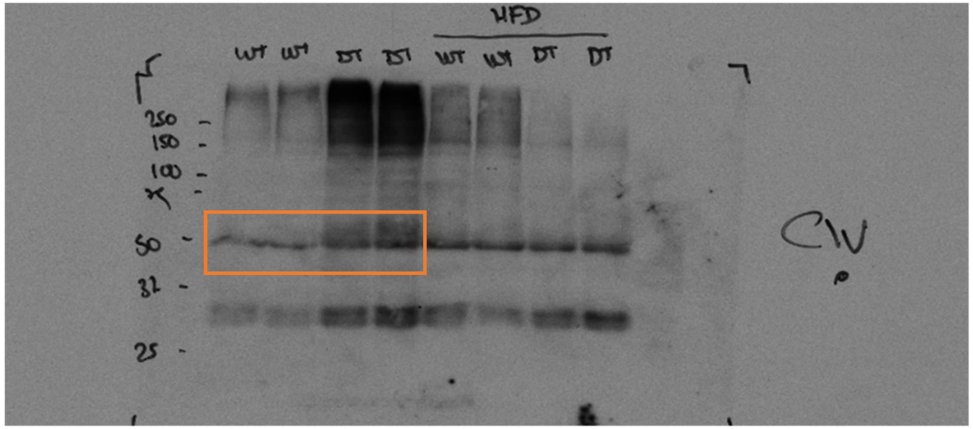

- **Figure 4K**
- <u>Ab: CIV</u>
- Date: 14/04/2019

wt wt dt dt wt wt dt dt

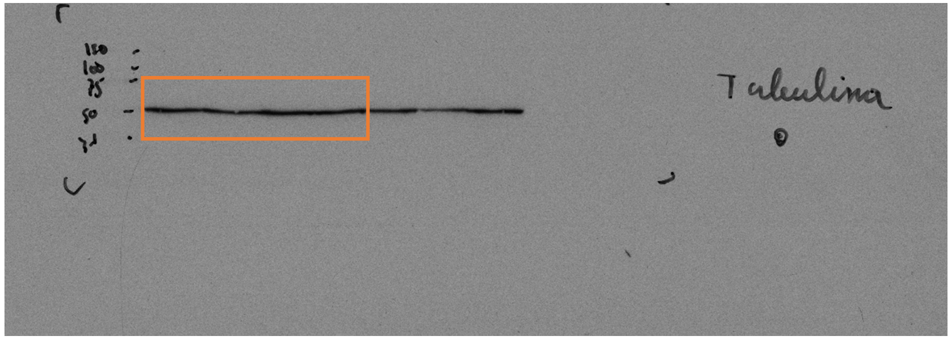

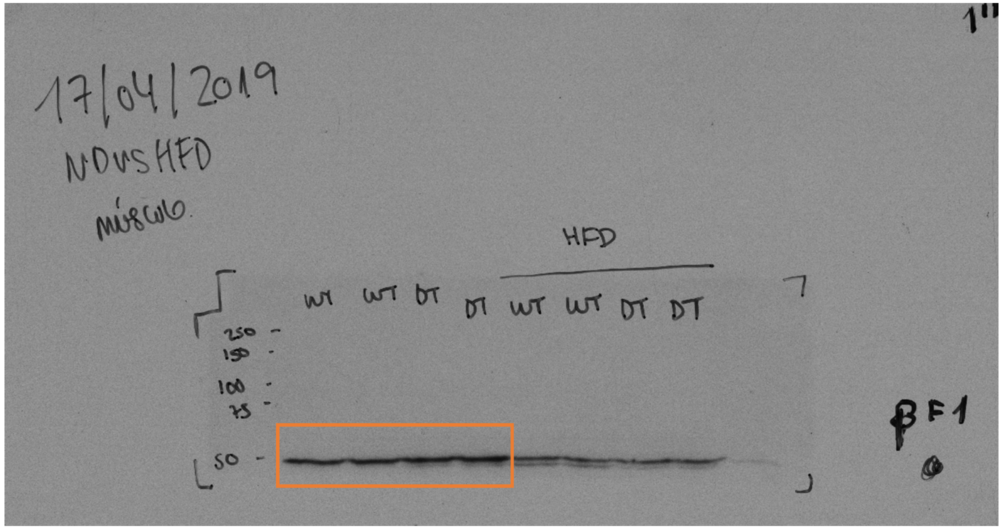

- **Figure 4K**
- <u>Ab: BF1</u>
- Date: 17/04/2018

- **Figure 4K**
- <u>Ab: tubulina</u>
- Date: 18/04/2018

Wt= wt
$ATPIF1_{H49K}$= DT
