# Supplementary material for: Dysfunctional oxidative phosphorylation shunts branched‐chain amino acid catabolism onto lipogenesis in skeletal muscle
Source: EMBO J. 2020 Jun 3;39(14):e103812. doi: 10.15252/embj.2019103812 (PMC7360968; doi:10.15252/embj.2019103812)
Supplement: Supplementary file 10 — Source Data for Figure 5 [file EMBJ-39-e103812-s008.pdf]

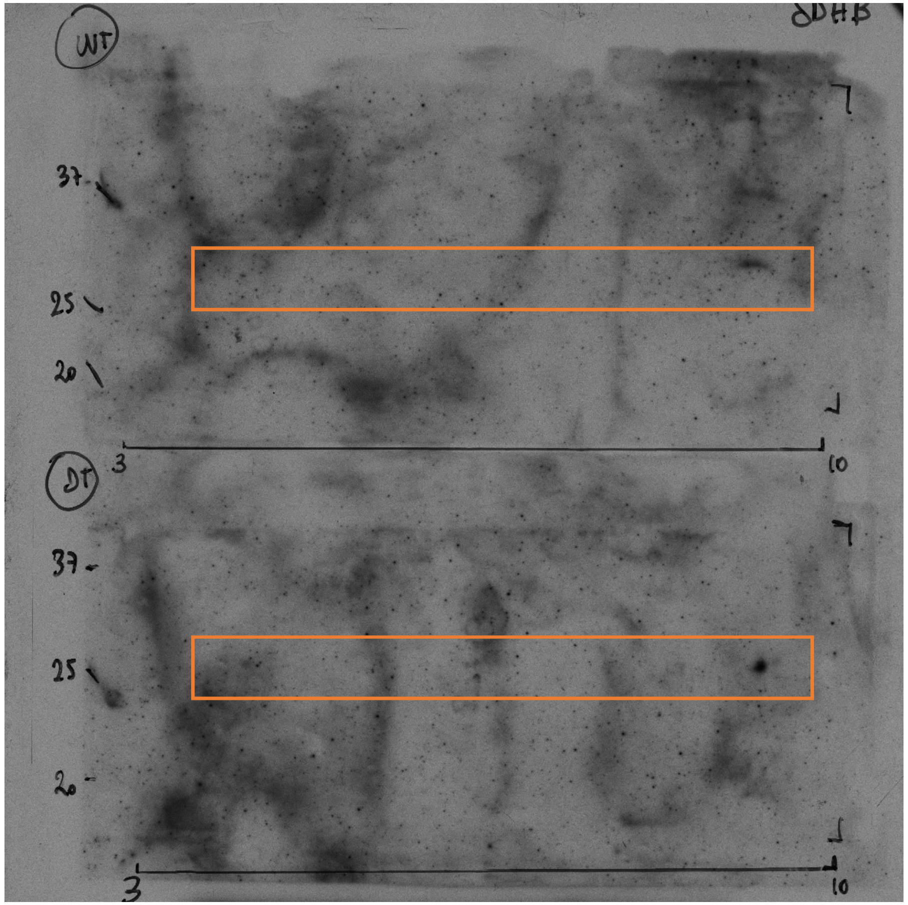

- **Figure 5C**
- Ab: SDH-B
- Date: 03/06/2019

- **Figure 5D**
- Ab: SDH-A
- Date: 03/06/2019

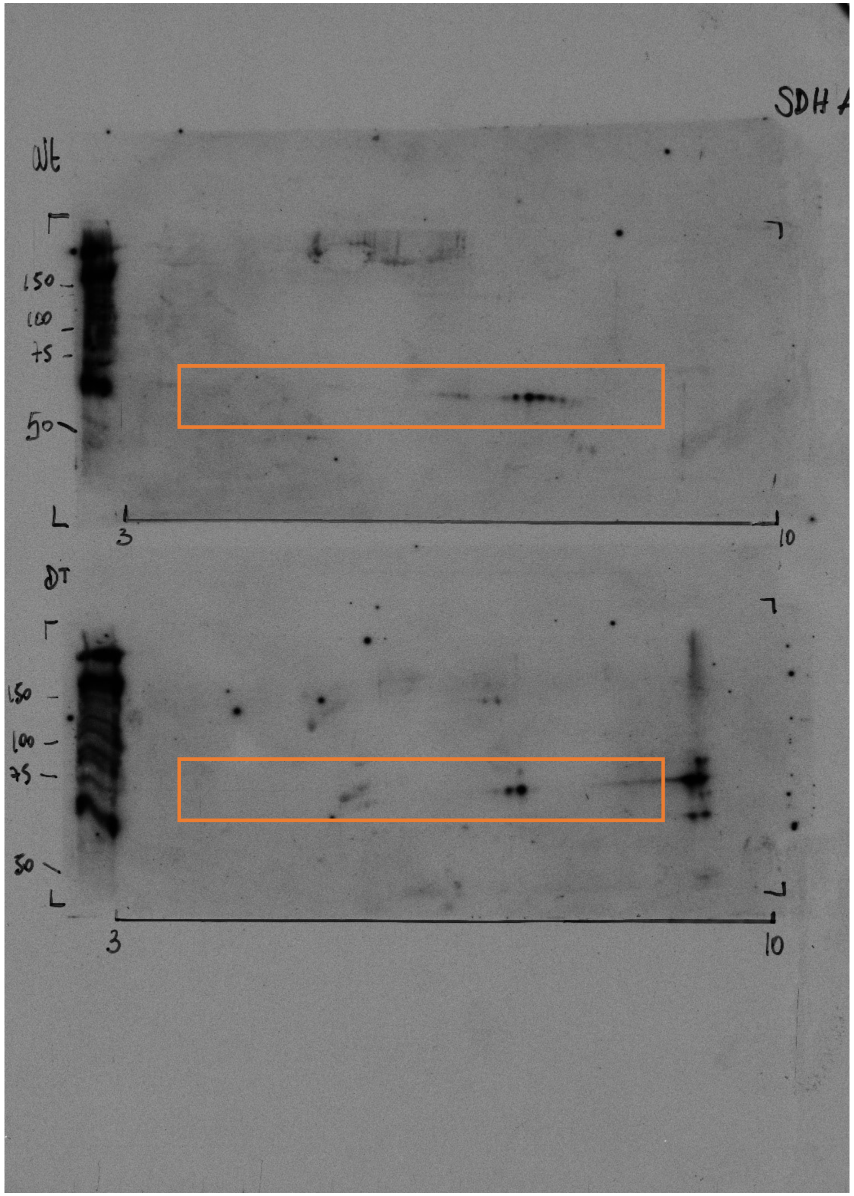

Wt= wt
$ATPIF1_{H49K}$= DT

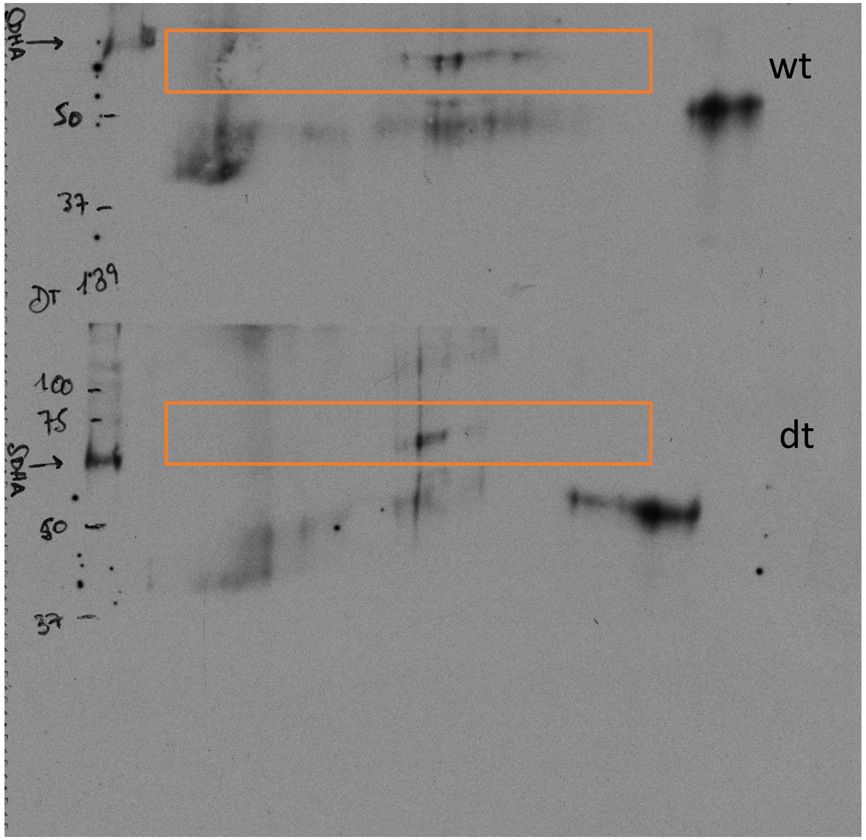

- **Figure 5E**
- Ab: SDH-A
- Date: 03/06/2019

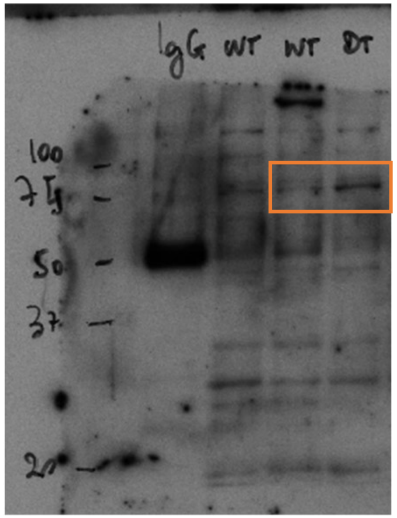

- **Figure 5F (1)**
- IP SDH-A,
- blot Acetyl-K
- Date: 18/06/2019

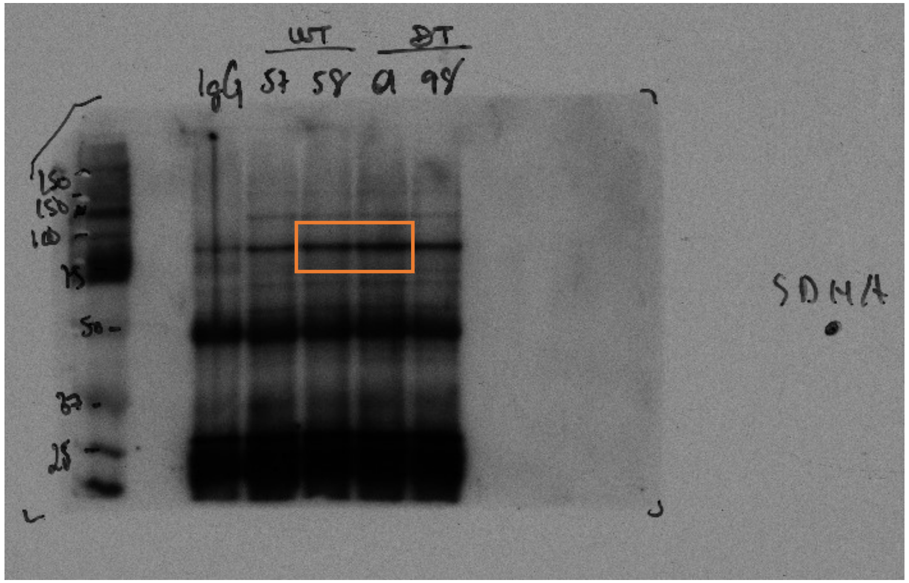

- **Figure 5F (3)**
- Ac: IP SDH-A, blot SDHA
- Date: 18/06/2019

- **Figure 5F (2)**
- IP Acetyl-K, blot SDH-A
- Date: 26/08/2019

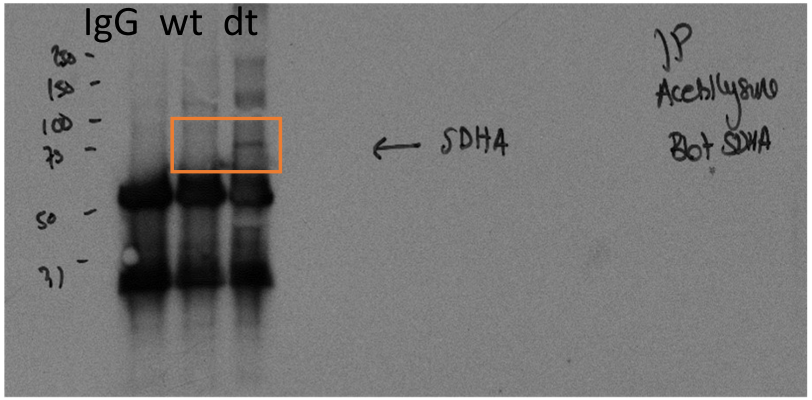

Wt= wt
$ATPIF1_{H49K}$= DT

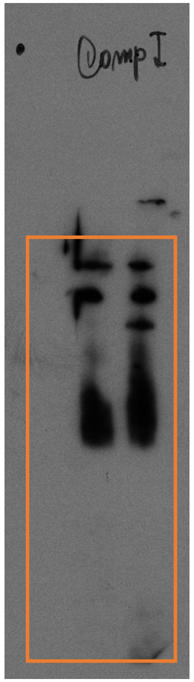

- **Figure 5K**
- Ab: CI
- 21/01/2019

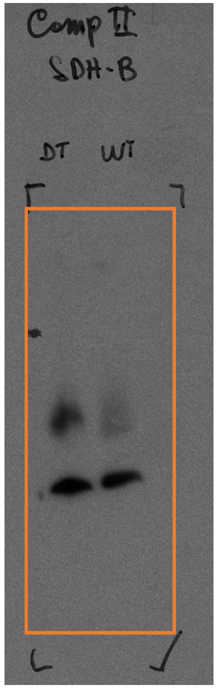

- **Figure 5K**
- Ab: CII (SDH-A)
- 21/01/2019

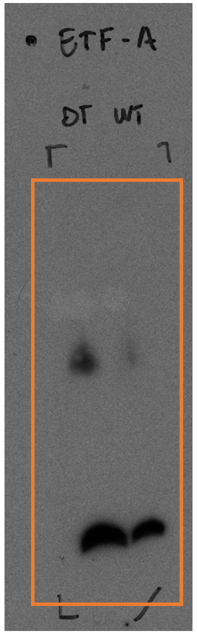

- **Figure 5K**
- Ab: ETFA
- 21/01/2019

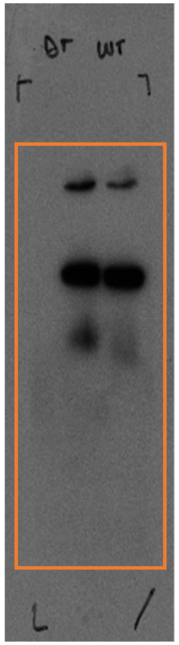

- **Figure 5K**
- Ab: Core II (CIII)
- 21/01/2019

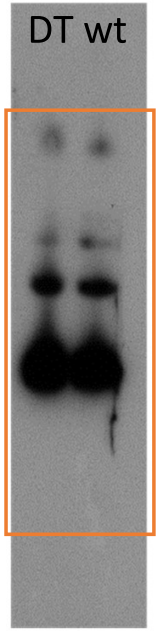

- **Figure 5K**
- Ab: CIV
- 21/01/2019

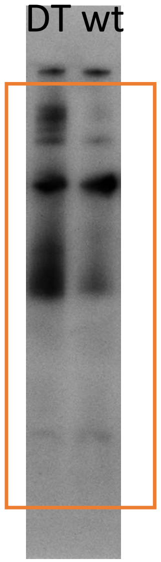

- **Figure 5K**
- Ab: BF1
- 29/01/2019

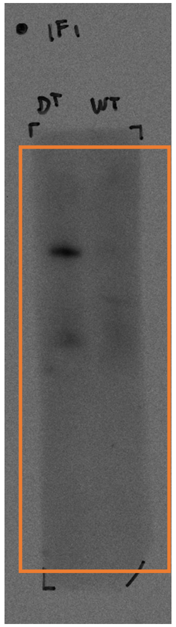

- **Figure 5K**
- Ab: IF1
- 29/01/2019

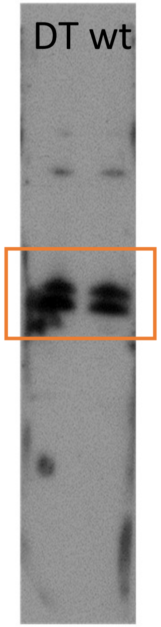

- **Figure 5K**
- Ab: VDAC
- 29/01/2019

Wt= wt
ATPIF1$_{H49K}$= DT

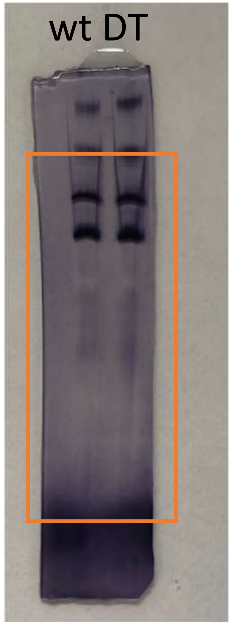

- **Figure 5K**
- Complex I activity
- 30/01/2019

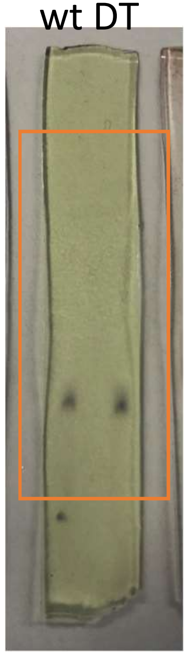

- **Figure 5K**
- Complex II activity
- 30/01/2019

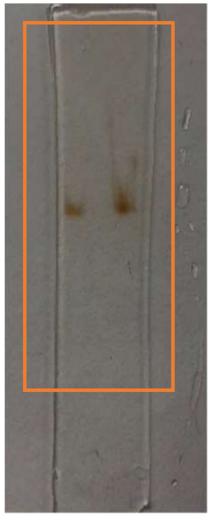

- **Figure 5K**
- Complex III activity
- 30/01/2019

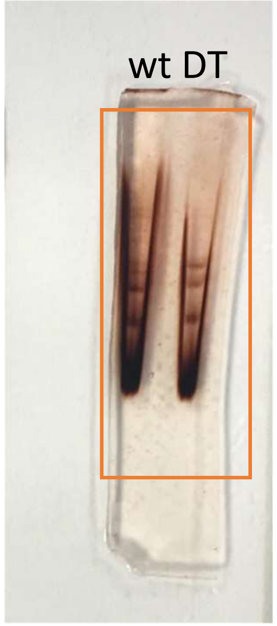

- **Figure 5K**
- Complex IV activity
- 30/01/2019

Wt= wt
ATPIF1$_{H49K}$= DT
