# Supplementary material for: Dysfunctional oxidative phosphorylation shunts branched‐chain amino acid catabolism onto lipogenesis in skeletal muscle
Source: EMBO J. 2020 Jun 3;39(14):e103812. doi: 10.15252/embj.2019103812 (PMC7360968; doi:10.15252/embj.2019103812)
Supplement: Supplementary file 11 — Source Data for Figure 7 [file EMBJ-39-e103812-s009.pdf]

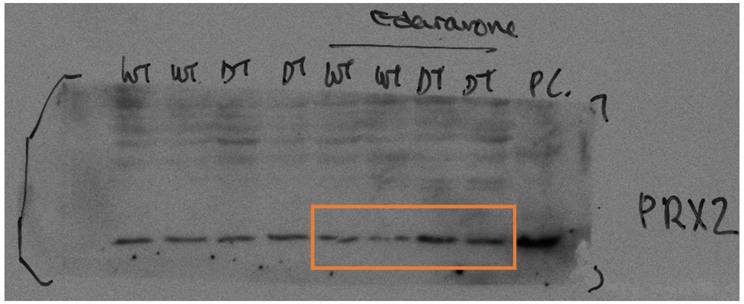

- **Figure 7B**
- <u>Ab: PRX2</u>
- Date: 12/02/2019

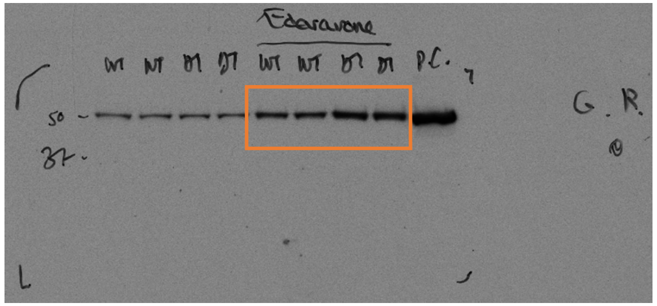

- **Figure 7B**
- <u>Ab: GSR</u>
- Date: 26/02/2019

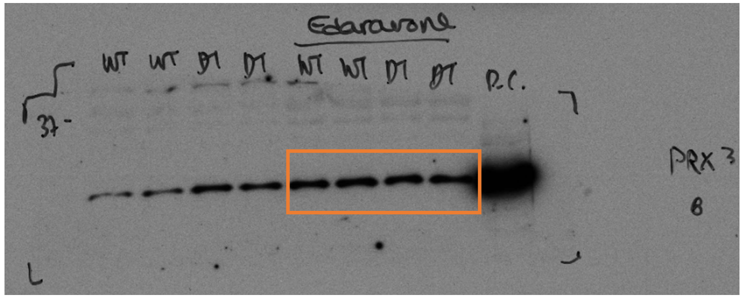

- **Figure 7B**
- <u>Ab: PRX3</u>
- Date: 26/02/2019

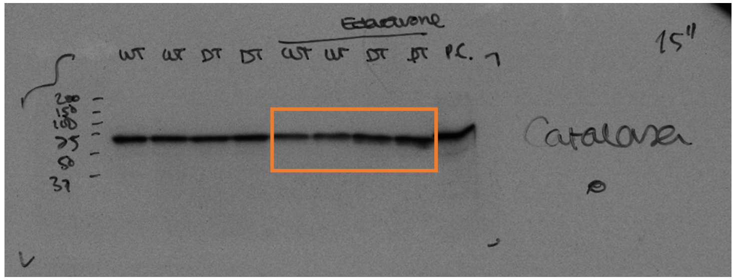

- **Figure 7B**
- <u>Ab: CATALASE</u>
- Date: 13/02/2019

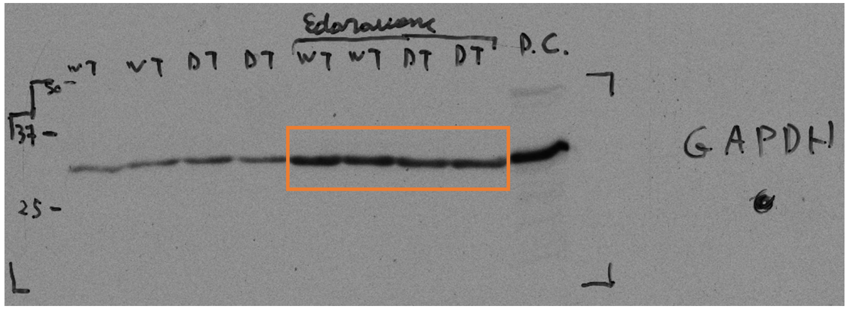

- **Figure 7B**
- <u>Ab: GAPDH</u>
- Date: 07/03/2019

Wt= wt
ATPIF1$_{H49K}$= DT

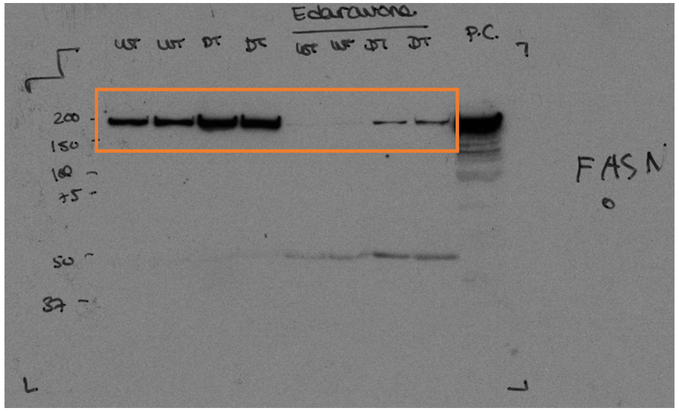

- **Figure 7D**
- Ab: FASN
- Date: 28/02/2019

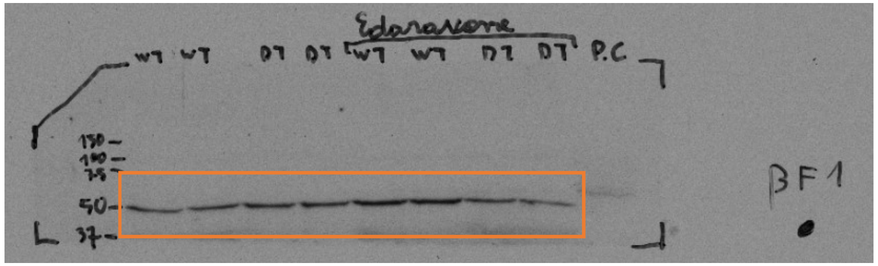

- **Figure 7D**
- Ab: BF1
- Date: 28/02/2019

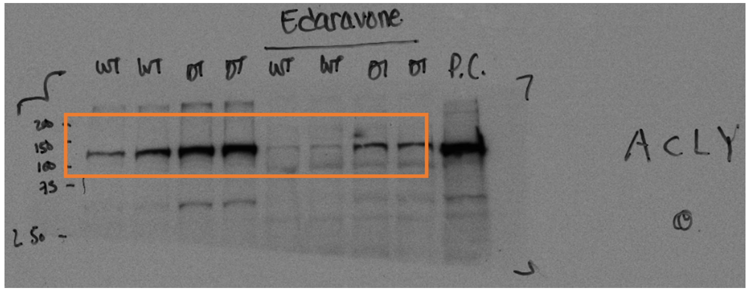

- **Figure 7D**
- Ab: ACLY
- Date: 26/02/2019

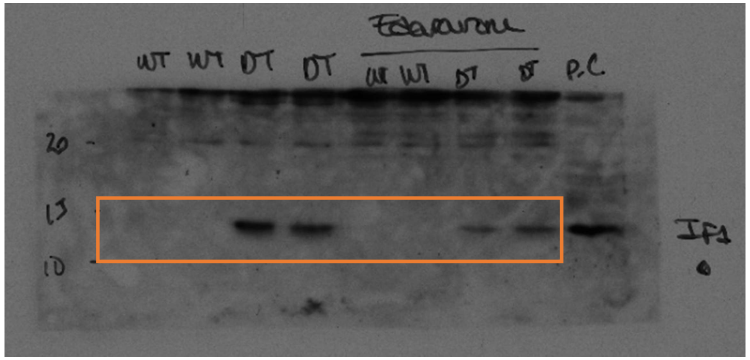

- **Figure 7D**
- Ab: human ATPIF1
- Date:28/02/2019

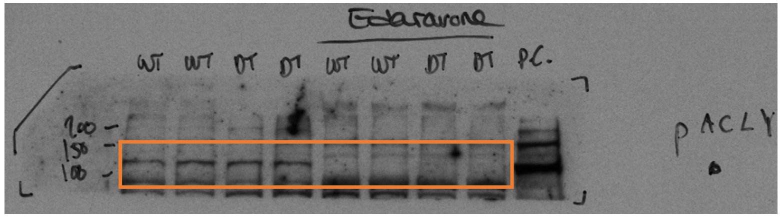

- **Figure 7D**
- Ab: pACLY
- Date: 26/02/2019

Wt= wt
$ATPIF1_{H49K}$= DT

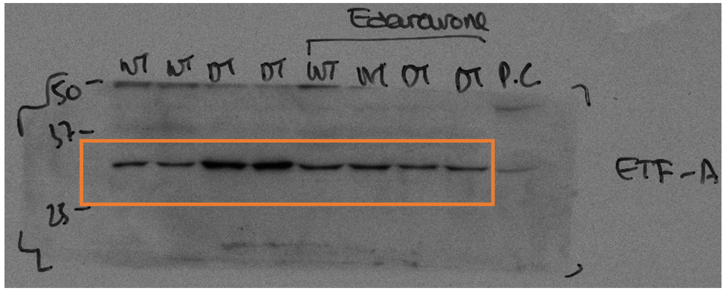

- **Figure 7E**
- Ab: ETF-A
- Date: 12/02/2019

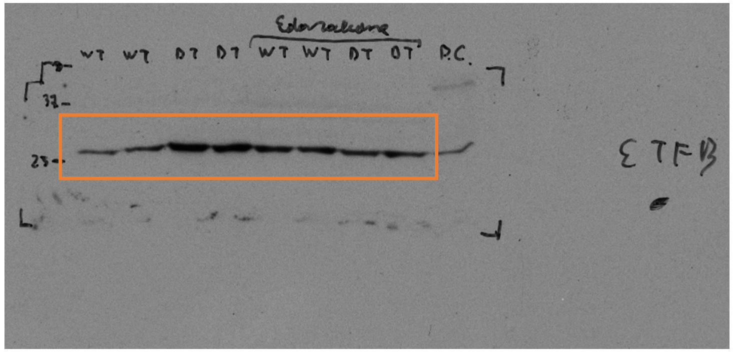

- **Figure 7C**
- Ab: ETF-B
- Date: 05/03/2019

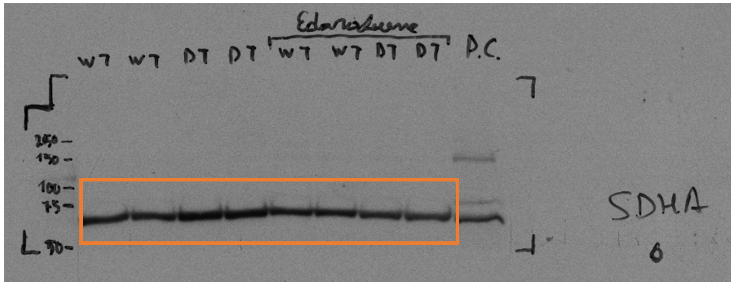

- **Figure 7C**
- Ab: SDH-A
- Date:05/03/2019

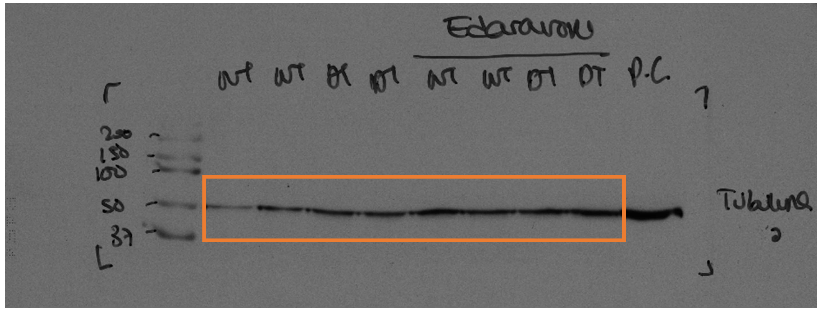

- **Figure 7C**
- Ab: Tubulin
- Date: 12/02/2019

Wt= wt
ATPIF1$_{H49K}$= DT

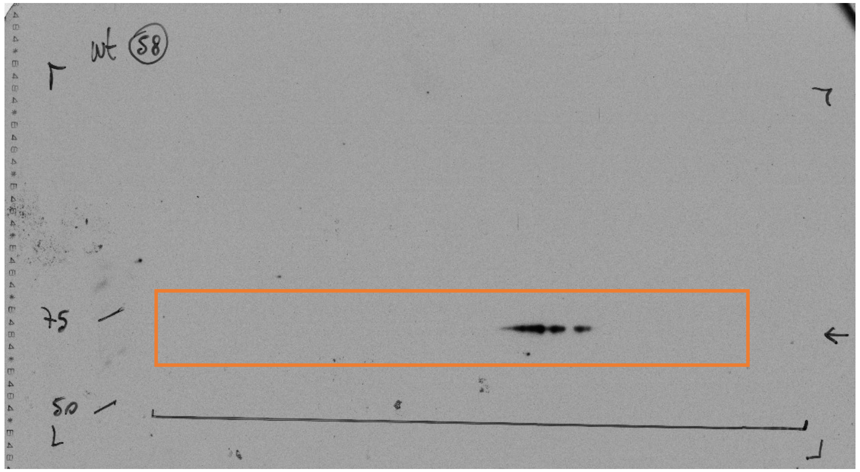

- **Figure 7F**
- Ab: SDHA
- Date: 21/02/2019

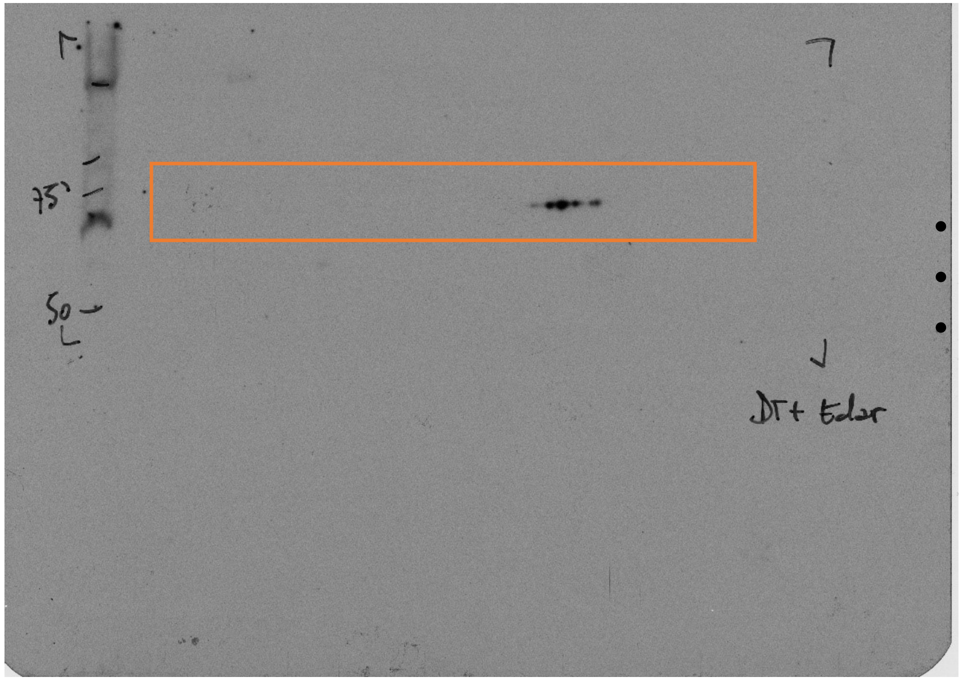

- **Figure 7F**
- Ab: SDHA
- Date:05/03/2019

Wt= wt
$ATPIF1_{H49K}$= DT

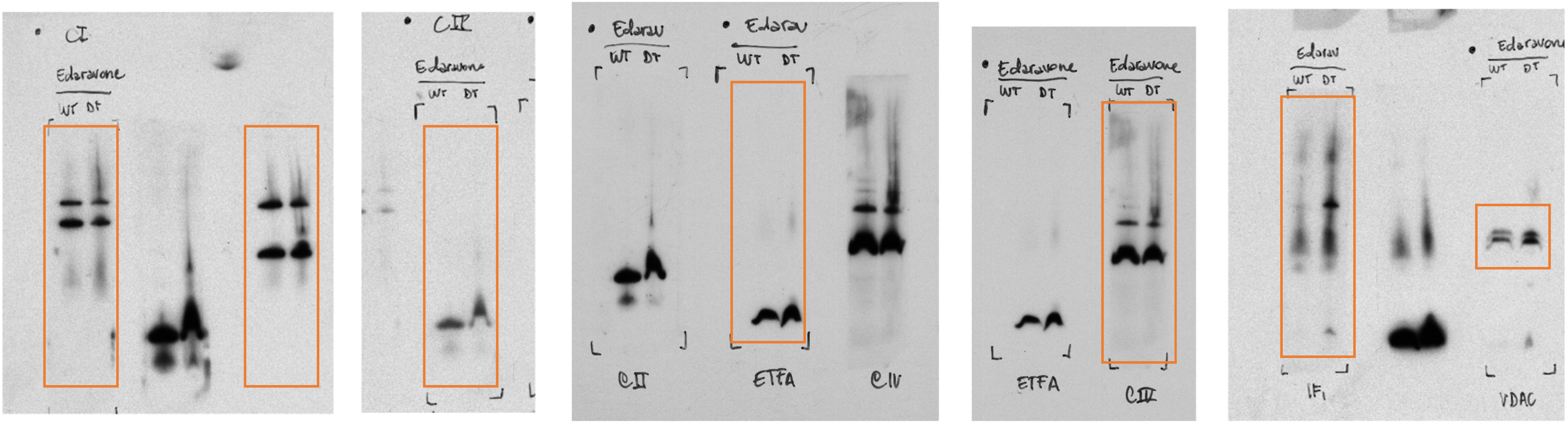

- **Figure 7G**
- Ab: CI
- 13/02/2019

- **Figure 7G**
- Ab: Core 2 (CIII)
- 13/02/2019

- **Figure 7G**
- Ab: CII
- 13/02/2019

- **Figure 7G**
- Ab: ETFA
- 13/02/2019

- **Figure 7G**
- Ab: CIV
- 13/02/2019

- **Figure 7G**
- Ab: ATPIF1
- 13/02/2019

- **Figure 7G**
- Ab: VDAC
- 13/02/2019

Wt= wt
$ATPIF1_{H49K}$= DT
